# Type VII secretion system gene mutations driving global mycobacterium tuberculosis transmission revealed by whole genomic sequence

**DOI:** 10.3389/fcimb.2025.1573643

**Published:** 2025-06-18

**Authors:** Jian-Jun Yang, Yuan-long Hu, Ping-yi Sun, Ling Wang, Xian-Jin Xie, Ting-Ting Wang

**Affiliations:** ^1^ Shandong University of Traditional Chinese Medicine, Jinan, China; ^2^ Shandong Provincial Third Hospital, Shandong University, Jinan, Shandong, China; ^3^ College of Integrated Chinese and Western Medicine, Jining Medical University, Jining, China; ^4^ Intensive Care Unit, People’s Hospital of Huaiyin Jinan, Jinan, China

**Keywords:** Mycobacterium tuberculosis, mutation, ESX, transmission, phylogenetic analysis

## Abstract

Pathogenic mycobacteria are able to transfer virulence factors across their complex cell wall using a type VII secretion system (T7SS)/early secreted antigenic target-6 of the kDa secretion system (ESX). Since the discovery of ESX loci during the Mycobacterium tuberculosis H37Rv genome project, extensive research in areas such as structural biology, cell biology, and evolutionary analysis has improved our understanding of the role of these systems. However, regulatory mechanisms for ESX in Mycobacterium tuberculosis remain elusive. Despite extensive research, the effects of ESX gene mutations on the dynamics of Mycobacterium tuberculosis transmission are not well understood. In this study, we investigated the role of ESX mutations in TB transmission, assessing their risk and characteristics. We analyzed 13582 whole genome sequences of Mycobacterium tuberculosis isolates, of which 6130 (45.13%) were clustered strains. Initially, Boruta algorithm was used to pinpoint SNPs that were significant for TB transmission. These SNPs were then subjected to univariate and multivariate logistic regression analysis to determine the significance of each SNP. The intersection of these two independent methods was recognized as the optimal set of risk mutations for TB transmission. Specifically, we identified one risk mutation (espA(Rv3616c, 4055801)) in L1, four risk mutations (espK(Rv3879c, 4357597), esxU(Rv3445c, 3863138), esxO(Rv2346c, 2626018), and esxW(Rv3620c, 4060588)) in L2, and four risk mutations (eccE1(Rv3882c, 4362807), espE(Rv3864, 4340330), espA(Rv3616c, 4055993), and eccC5(Rv1783, 2019942)) in L4. These risk mutations were significantly associated with clustering, potentially increasing TB transmission. Our findings suggest that mutations in ESX genes play a crucial role in Mycobacterium tuberculosis transmission. These results can be applied to the development of novel strategies for the treatment and prevention of disease.

## 1 Introduction

Tuberculosis (TB) is a widespread infectious disease caused by a pathogen known as Mycobacterium tuberculosis. In recent years, TB cases have decreased worldwide, but the number of people infected with TB each year is still high. In 2022, 10 million people will be infected with TB and 1.3 million will die from TB, causing TB, along with HIV/AIDS. Mycobacteria are adept at evading the host’s immune system and establishing infection by engaging with host factors and secreting a range of protein families, including Esx, Esp, and PE/PPE, to exploit the host’s nutrients and evade destruction by the immune system (Gröschel et al., 2016; Tufariello et al., 2016; Ates, 2020; Rivera-Calzada et al., 2021). The Mycobacterium tuberculosis genome encodes five specialized secretion systems, referred to as ESX or type VII systems, termed ESX-1 to ESX-5 (Cole et al., 1998). Mycobacterial virulence factors are typically defined as bacterial genes or cellular components that enable their overall survival in the host (Ly and Liu, 2020). ESX systems as well-established virulence factors (Ly and Liu, 2020) are multisubunit apparatuses that have similar structures and secrete related proteins which play a key role in mycobacterial proliferation, pathogenesis, cytosolic escape within macrophages, regulation of macrophage apoptosis, metal ion homeostasis, etc (Majlessi et al., 2015; van Winden et al., 2019; Bunduc et al., 2020a; Famelis et al., 2023). TB transmission is influenced by various factors such as human behavior, virulence of the Mycobacterium tuberculosis pathogen, and host immune responses. Numerous animal and immunological experiments have been carried out using ESX systems to investigate their significance in mycobacterial virulence. These studies indicate that ESX systems play a crucial role in mycobacterial pathogenesis (de Jonge et al., 2007; MacGurn and Cox, 2007; van der Wel et al., 2007; Smith et al., 2008; Sani et al., 2010; Abdallah et al., 2011; Houben et al., 2012; Simeone et al., 2012; Watson and Cox, 2012; Champion et al., 2014; Pajuelo et al., 2021). Thus, variations in ESX systems gene across Mycobacterium tuberculosis lineages may account for differences in TB transmissibility. Here we compared SNPs in the ESX gene region between “clustered” and “non-clustered” isolates in different lineages to identify the role of ESX mutations in TB transmission combining whole genome sequence (WGS) data from 13582 global Mycobacterium tuberculosis isolates collected from 1984-2018.

In recent years, whole genome sequencing (WGS) studies of Mycobacterium tuberculosis have significantly expanded our understanding of this notorious pathogen. The first genome of Mycobacterium tuberculosis was published in 1998, and WGS has since provided a more comprehensive overview of the genomic features of Mycobacterium tuberculosis, identifying specific mutations that help Mycobacterium tuberculosis reduce immune surveillance and drug treatment capabilities. This study used WGS to evaluate the influence of ESX-related gene mutations on the transmission of Mycobacterium tuberculosis and clustering was used to represent the transmission chain of Mycobacterium tuberculosis (Rodríguez et al., 2010).

## 2 Materials and methods

### 2.1 Clinical isolates and whole-genome sequencing

**Figure 1:**
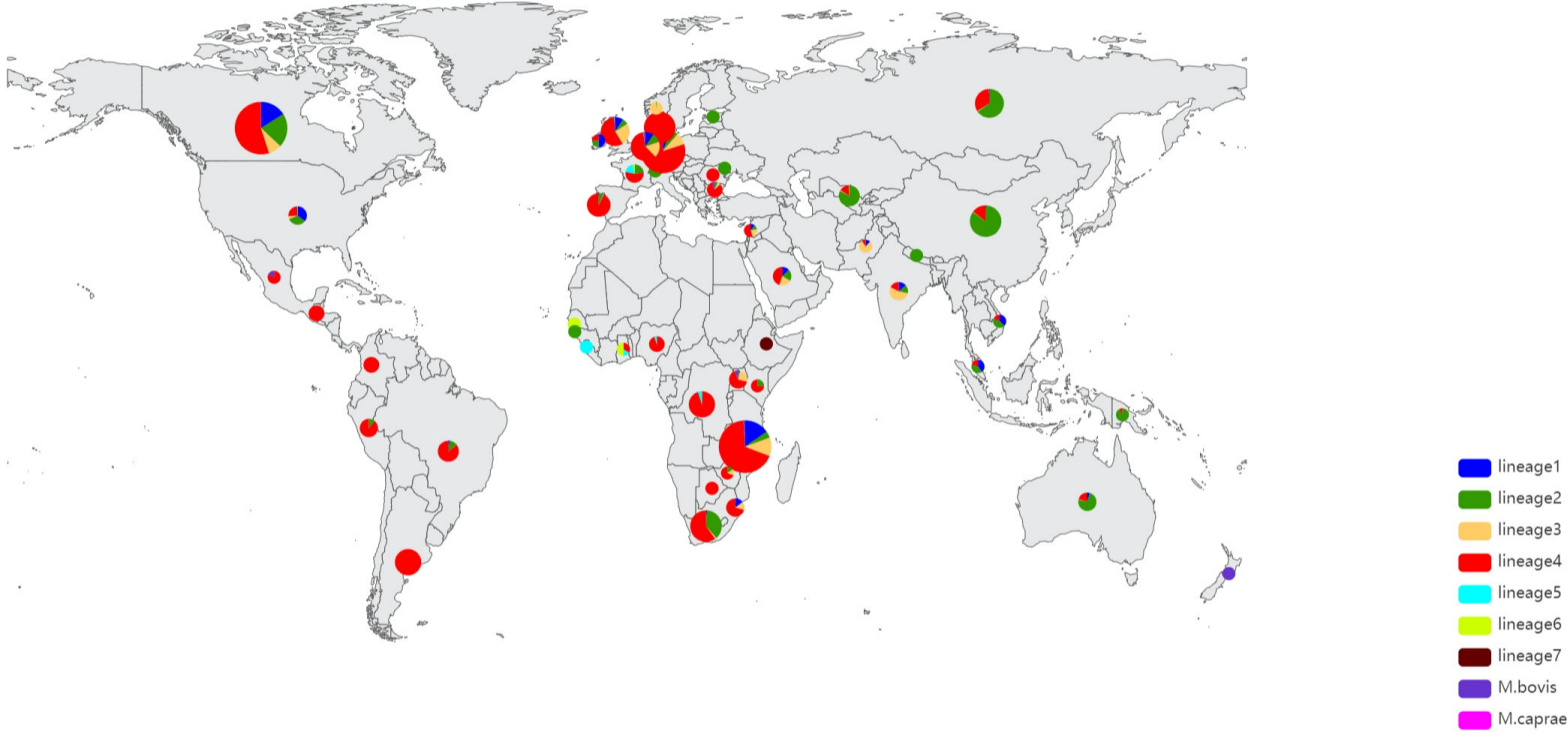
Sample size and lineages proportion in different countries of the 13858 isolates.

We extracted genomic DNA using Cetyltrimethylammonium Bromide (CTAB) from 1468 Mycobacterium tuberculosis samples from Shandong Province over a 5-year period, and 1445 samples passed Quality control(QC). QC was performed using FastQC software to ensure the quality of sequenced reads. The genomes were sequenced using the Illumina HiSeq 4000 system. We also used public databases to compile a global collection of clinical isolates of Mycobacterium tuberculosis, ensuring a diverse and representative collection of genomes with the broadest geographic coverage possible (Luo et al., 2015; Yang et al., 2017; Coll et al., 2018; Hicks et al., 2018; Koster et al., 2018; Liu et al., 2018; Chen et al., 2019; Huang et al., 2019; Jiang et al., 2020). The isolate metadata were downloaded using SRAtools v2.9.1 (https://github.com/ncbi/sra-tools). Only the genomes annotated with sampling date and country of origin were included in the present study. The dataset includes newly sequenced dataset of 1445 Mycobacterium tuberculosis strains and the 12413 Mycobacterium tuberculosis strains collected from 50 countries, a total of 13858 Mycobacterium tuberculosis of isolates. Among the 13858 Mycobacterium tuberculosis isolates, China contributed the most isolates (3408), while Gambia and Moldova contributed the least (1 each). Botswana, Guinea-Bissau, and Sierra-Leone contributed 2 isolates each, Ireland contributed 4 isolates, Switzerland and Malaysia contributed 5 isolates, Mexico, Ghana, and Estonia contributed 6 isolates each, South Africa, Nepal, and Kenya contributed 8 isolates each, Romania and Lebanon contributed 9 isolates each, and several other countries or regions contributed between 11 and 1650 isolates. (**Figure 1**) We utilized BWA-MEM (version 0.7.17-r1188) to accurately map the reference genome of the standard isolate Mycobacterium tuberculosis H37Rv. Our analysis only included samples exhibiting a coverage rate of 98% or higher and a minimum depth of at least 20% (Jung and Han, 2022). Finally, a total of 13582 genomes were analyzed, please refer to **Additional File 1**: **Supplementary Tables S1, S2** for the specific sample numbers.

### 2.2 SNP identification

Variant calling was performed using Freebayes (version 1.3.2) and bcftools (version 1.15.1) with a filter parameter ‘FMT/GT=“1/1” && QUAL>=100 && FMT/DP>=10 && (FMT/AO)/(FMT/DP)>=0’. Single nucleotide polymorphisms in previously defined repetitive regions were excluded, including PPE and PE-PGRS genes, and mobile elements or repeat regions and repeat bases generated by TRF (version 4.09) and Repeatmask (version 4.1.2-p1) (Benson, 1999; Saha et al., 2008; Garrison and Marth, 2012; Danecek et al., 2021). Finally, SNP annotation was conducted via SnpEff v 4.1 l, with the resulting output obtained utilizing the Python programming language (Cingolani et al., 2012). Genotypic drug resistance of each isolate was predicted in TBProfiler using an established library of mutations (https://github.com/jodyphelan/tbdb) (Coll et al., 2015). The virulence factor database (http://www.mgc.ac.cn/VFs/) contains various medically important bacterial pathogen virulence factors, which include 86 experimentally confirmed and 171 putative genes related to the virulence of Mycobacterium tuberculosis (Cole et al., 1998; Liu et al., 2022). Python was utilized to detect mutations in genes associated with ESXs (**Additional File 1**: **Supplementary Table S3**). FASTA sequences of these genes were used to search for corresponding genes in the 12 genomes using BLAST.

### 2.3 Mtb lineage and genomic cluster

We used the web-based tool TBProfiler (version 4.3.0) to analyze 13858 Mycobacterium tuberculosis WGS data to assign lineages and predict drug resistance (**Additional File 1**: **Supplementary Table S4**) (Coll et al., 2014; Phelan et al., 2019). Genomic clusters were ascertained independently of the epidemiological data, and Genomic clusters were inferred based on how genetically similar two isolates were from each other. The upper thresholds of genomic relatedness or cluster is defined as 12 SNPs or alleles cut off or less and a recent transmission event is defined as 5 or less SNPs or alleles (Walker et al., 2013; Kohl et al., 2018). In this study Mycobacterium tuberculosis isolates with a genomic difference (s) ≤ 12 single nucleotide polymorphisms (SNPs) were defined as a genomic cluster (Yang et al., 2017) for further analysis of transmission cluster to avoid missing cases and incorporating recent and old transmission events, which is similar to definitions used in previous genomic studies of Mycobacterium tuberculosis transmission (Walker et al., 2013; Walker et al., 2014; Holt et al., 2018). As suggested by recent analysis of intra-patient variation, the estimate of 5 SNPs may be too low (Lieberman et al., 2016), we finally chose the cut-off of 12 SNPs to define transmission clusters for further analysis based on the previous study (Walker et al., 2013; Walker et al., 2014; Holt et al., 2018). Additionally, according to the classification of transmission clusters by scholars, we also divided transmission clusters into large, medium, or small (large, over 9 isolates; medium, between 3 and 9 isolates; and small, 2 isolates).

### 2.4 Phylogenetic analysis

**Figure 2:**
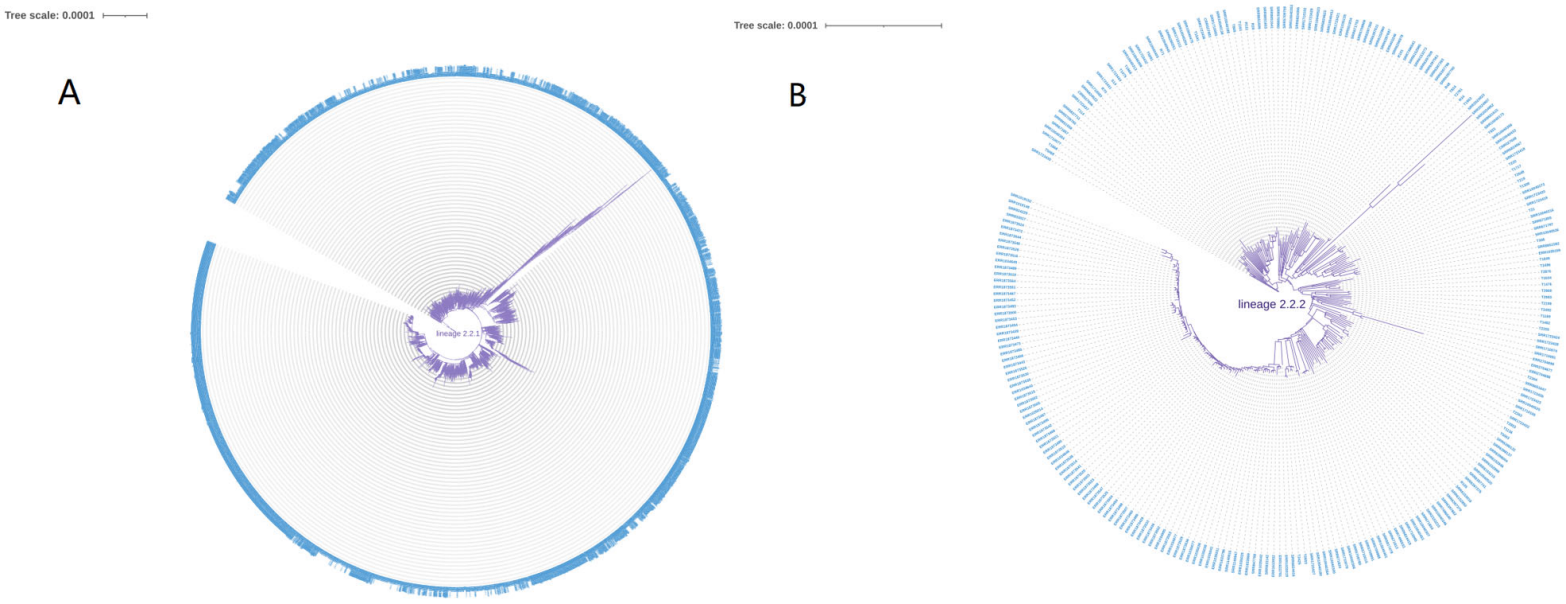
**(A)** The phylogenetic tree analysis of lineage1.1. **(B)** The phylogenetic tree analysis of lineage1.2.

Reference genome with only substitution variants instantiated was used as the sample’s genome. Maximum-likelihood (ML) phylogenetic trees were constructed and dated by IQ-TREE (v1.6.12) model “JC+I+G4” with 1000 ultrafast bootstrap replicates and treetime (v0.9.0) [GitHub - neherlab/treetime: Maximum likelihood inference of time stamped phylogenies and ancestral reconstruction (Zelner et al., 2016) https://github.com/neherlab/treetime. The trees were constructed using the highest likelihood model selected by automatic model selection in IQ-TREE (v1.6.12), which utilized the JC model of nucleotide substitution and invariable site plus discrete Gamma model of rate heterogeneity to analyze the genome samples with only substitution variants replaced in reference sequence. The resultant phylogenetic tree was visualized through the utilization of iTOL (Letunic, 2021) (https://itol.embl.de/). A maximum likelihood phylogenetic tree was constructed for lineage 1 as shown in **Figure 2**. Additional tree analysis for lineages 2–7 is available in **Additional File 2**: **Supplementary Figures S2–S7**.

### 2.5 Independent risk mutations selection

SNPs were found to be associated with clustering, which may potentially enhance TB transmission. These SNPs are referred to as risk mutations. A Boruta algorithm was used in R (version 4.3.0, Boruta package) to select independent risk mutations for TB transmission. The response variable is whether the bacterial strain is clustered or not. The features are these SNPs. The Boruta algorithm has proven to be effective in over 100 studies and is recognized as a premier tool for evaluating large datasets (Saulnier et al., 2011; Degenhardt and Szymczak, 2019). It is a feature selection algorithm based on a random forest classifier (Kursa, 2010; Kursa and Rudnicki, 2010; Degenhardt and Szymczak, 2019). Unlike a general feature selection algorithm, the Boruta feature selection algorithm aims to select the set of features that are most relevant to the dependent variable rather than to a particular model. The Boruta algorithm produces three outcomes for input features, which include confirmed features, tentative features, and rejected features. Thus, the independent risk mutations in this study were screened by the Boruta algorithm.

### 2.6 Univariate and multivariate/ordinal logistic regression analysis

To verify selected mutations with the Boruta algorithm, all mutations were estimated using univariate and multivariate/ordinal logistic regression analysis. We compared SNPs in the ESX gene region between “clustered” and “non-clustered” isolates using univariate and multivariate/ordinal logistic regression analysis in different lineages.

### 2.7 Statistical analysis

All statistics were performed with SPSS (version 26) and R software (version 4.2.0). Factors with a P-value less than 0.05 in the final model were considered to be independently associated with genomic clusters. The odds ratios (OR) and 95% confidence intervals (95% CI) were calculated. Due to the limited sample size, we analyzed only the isolates of lineages 1, 2, 3, and 4, excluding the remaining lineages for this study. In addition, in a thorough examination of 481 strains from Denmark (**Additional File 1**: **Supplementary Table S5**), we discovered that only twenty SNP variations were present in the ESX gene region, a stark contrast to the plethora of SNPs found in other strains. Therefore, we excluded these 481 Danish strains from our subsequent analysis. We just analyzed nonsynonymous sites and those sites with a mutation frequency higher than 0.02 in lineage1,2,3 and 4. There are 38,16,24 and 26 mutation sites in lineage1,2,3 and 4 respectively, totally 104 mutation sites. The mutation frequency was calculated as the percentage of mutation isolates among total isolates in different lineages (number of mutation isolates/number of total isolates in different lineages). In terms of SNPs, isolates that possess the SNP in the ESX gene region are referred to as mutation isolates.

### 2.8 Predicted impact of mutations on proteins

Protein prediction algorithm, I-Mutant v2.0 (http://folding.biofold.org/i-mutant/i-mutant2.0.html), was used to predict the functional impact of noteworthy SNPs on protein structure and function.

### 2.9 Genomic data availability

The newly sequenced whole genome dataset of 1,445 M. tuberculosis strains was deposited in the National Center for Biotechnology Information (NCBI) under BioProject PRJNA1002108 and 12137 other isolates were downloaded from the public databases. For more details about the 13582 genomes, please consult **Additional File 1**: **Supplementary Tables S1, S2** containing the specific sample numbers. Additional data can be obtained by contacting the corresponding authors upon request.

## 3 Results

### 3.1 Sample description

All seven global lineages (L1-7) of Mycobacterium tuberculosis were detected. Amonge 13582 strains, 851 strains were classified as L1 (6.27%), 5136 strains were assigned to L2 (37.81%), 970 strains belonged to L3 (7.14%), 6489 strains to L4 (47.78%), and only 38 (0.28%), 10 (0.07%), 29 (0.21%), 55(0.40%), 1(0.00%) and 3(0.00%) isolates belonged to L5, 6, 7, M.bovis, M.orygis and M.caprae respectively. Most strains were sublineages 2.2.1 (n=4832, 35.58%), while the remaining sublineages contained less than 1919 strains. For further details, see **Table 1**.

**Table 1:** Characteristics of the 13582 M. tuberculosis.

Characteristic		Classification	No. (%)
**Lineage**	Lineage1		851(6.27)
	Lineage2		5136(37.81)
	Lineage3		970(7.14)
	Lineage4		6489(47.78)
	Lineage5		38(0.28)
	Lineage6		10(0.07)
	Lineage7		29(0.21)
	M.bovis		55(0.40)
	M.orygis		1(0.00)
	M.caprae		3(0.00)
**Sub-lineage**	Lineage2.1		46(0.34)
	Lineage2.2.1		4832(35.58)
	Lineage2.2.2		258 (1.90)
	Lineage4.1		1614(11.88)
	Lineage4.2		427(3.14)
	Lineage4.3		1919(14.13)
	Lineage4.4		626(4.61)
	Lineage4.8		1086(7.80)
	Other sub-L4		817(6.02)
**Clustered Strains**	Lineage1	Clustered strains	148(17.39)
		No-clustered strains	703(82.61)
	Lineage2	Clustered strains	2131(41.50)
		No-clustered strains	3004(58.50)
	Lineage3	Clustered strains	280(28.87)
		No-clustered strains	690(71.13)
	Lineage4	Clustered strains	3124(48.14)
		No-clustered strains	3365(51.86)
**Clustered strains _size**	Lineage1	Large clustered strains	0(0.00)
		Medium clustered strains	36(4.23)
		Small clustered strains	112(13.16)
		No-clustered strains	704(82.73)
	Lineage2	Large clustered strains	317(6.17)
		Medium clustered strains	797(15.52)
		Small clustered strains	1018(19.82)
		No-clustered strains	3004(58.50)
	Lineage3	Large clustered strains	10(1.03)
		Medium clustered strains	118(12.16)
		Small clustered strains	152(15.67)
		No-clustered strains	690(71.13)
	Lineage4	Large clustered strains	647(9.97)
		Medium clustered strains	1330(20.50)
		Small clustered strains	1148(17.69)
		No-clustered strains	2884(44.44)

### 3.2 Risk mutations associated with genomic clusters

**Figure 3:**
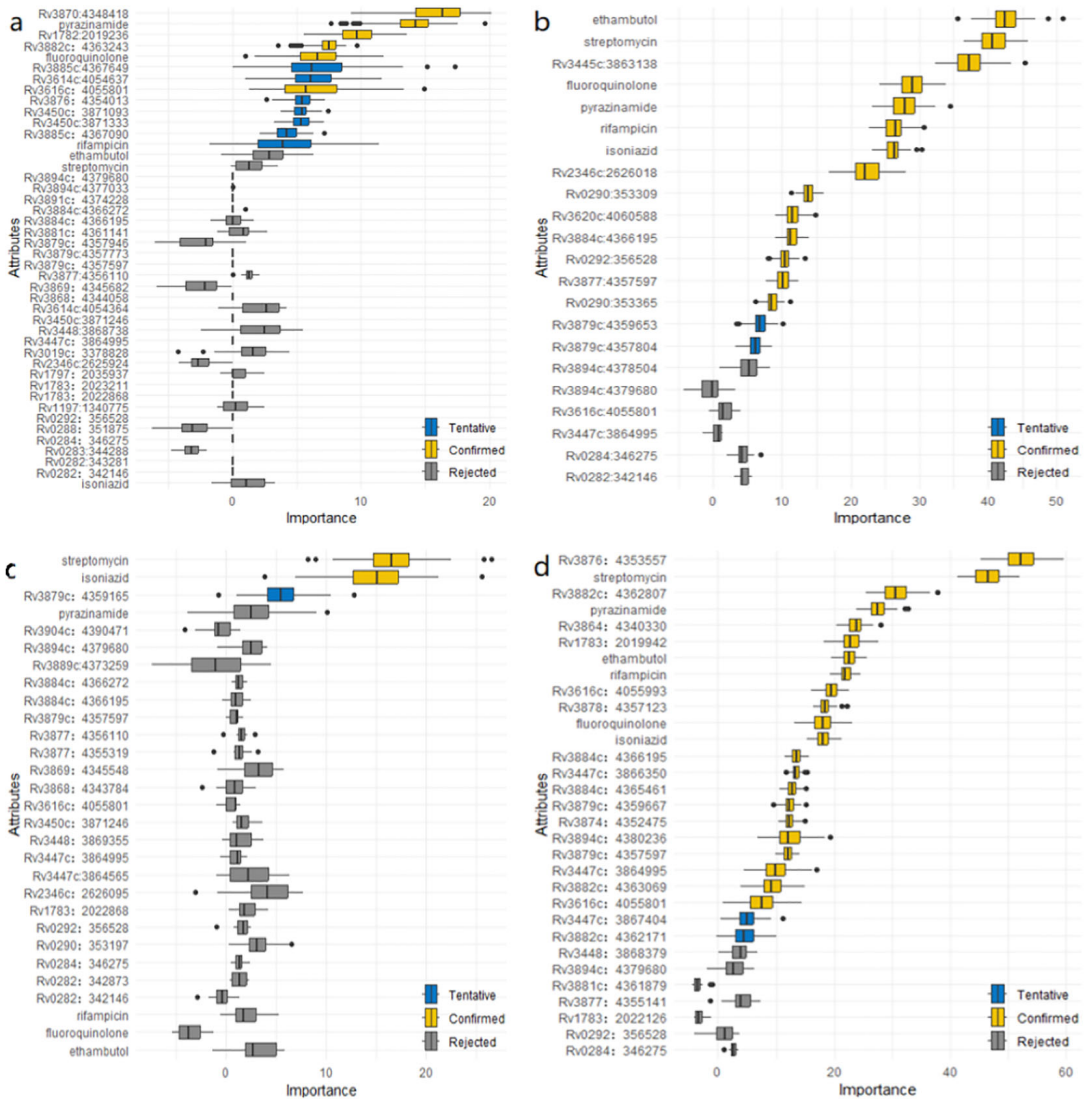
**(a)** Mutations associated with genomic clusters in ESX gene region of lineage 1 identified by the Boruta algorithm. **(b)** Mutations associated with genomic clusters in ESX gene region of lineage 2 identified by the Boruta algorithm. **(c)** Mutations associated with genomic clusters in ESX gene region of lineage 3 identified by the Boruta algorithm. **(d)** Mutations associated with genomic clusters in ESX gene region of lineage 4 identified by the Boruta algorithm.

**Figure 4:**
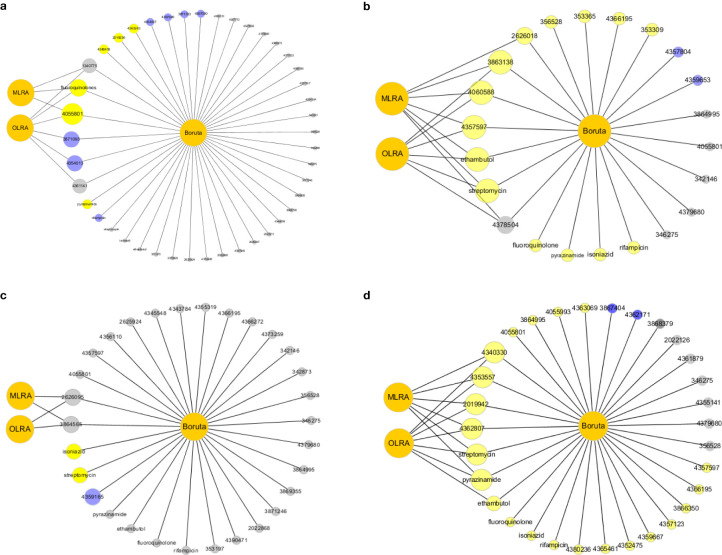
**(a)** The intersection results of Lineage 1. The grey color means reject in the Boruta algorithm. The blue color means tentative in the Boruta algorithm. The yellow color means confirm in the Boruta algorithm. MLRA was the abbreviation of Multivariate Logistic Regression Analysis. OLRA was the abbreviation of Ordinal Logistic Regression Analysis. If the circle connected with MLRA or OLRA, it means the SNPs were risk mutations in MLRA or OLRA. **(b)** The intersection results of Lineage 2. **(c)** The intersection results of Lineage 3. **(d)** The intersection results of Lineage 4.

In this study, we detected risk mutations associated with genomic clusters in the four lineages and sublineages of L2.2.1, L4.1, L4.3 and L4.8. The Boruta algorithm was used initially to identify mutations associated with genomic clusters (**Figure 3**; **Additional File 2**: **Supplementary Figures S8–S11**). Next, univariate and multivariate logistic regression analysis were performed to evaluate the significance of each selected mutations (**Additional File 1**: **Supplementary Tables S6-S15**). Finally, the intersection results of the two independent methods were identified as the optimal feature variables which were risk mutations associated with genomic clusters, and are summarized in **Figure 4**; **Additional File 2**: **Supplementary Figures S12-S15**. Specifically, there was one risk mutation[espA(Rv3616c, 4055801)] in L1, four risk mutations [esxU(Rv3445c, 3863138), esxO(Rv2346c, 2626018), esxW(Rv3620c, 4060588) and espK(Rv3879c, 4357597)] in L2, four risk mutations[espE(Rv3864, 4340330), espl (Rv3876, 4353557), eccC5(Rv1783, 2019942) and eccE1(Rv3882c, 4362807)] in L4. Three risk mutations [esxU(Rv3445c, 3863138), esxO(Rv2346c, 2626018) and eccA2(Rv3884c, 4366195)] in L2.2.1, one risk mutation [espK(Rv3879c, 4359667)]in L4.1 and one risk mutation [eccE1 (Rv3882c, 4362171)]in L4.8.

In addition, the intersection results in antimicrobial resistance mutations of the two independent methods were also identified in different lineages (**Figure 4**; **Additional File 2**: **Supplementary Figures S12–S15**). Antimicrobial resistance mutation at L1, L2, and L4 was determined to be associated with an elevated clustering risk. Specifically, a single mutation was noted for fluoroquinolones at L1, two mutations were found for streptomycin and ethambutol at L2, and two mutations were identified for pyrazinamide and streptomycin at L4. Three mutations (streptomycin, ethambutol and pyrazinamide) in L2.2.1, two mutations (pyrazinamide and streptomycin) in L4.1 and three mutations (fluoroquinolones, pyrazinamide and streptomycin) in L4.3 were associated with genomic clusters. Mutations occurred mainly in drug resistance genes such as katG, rpoB, rpsL, embB, pncA, gyrA, and ethA. Drug resistance is an important factor of TB transmission. In our study, we mainly used the Drug resistance mutations as exposure factors in multivariate logistic regression analysis to improve the sensitivity of analysis results.

#### 3.2.1 Risk mutations associated with genomic clusters selected by Boruta algorithm

By comparing original mutations (attributes) importance with importance achievable at random, the results of Boruta algorithm for L1, L2, L3 and L4 were shown in **Figure 3**.

In **Figure 3a**, six mutations (Rv3870:4348418, Rv1782:2019236, Rv3882c:4363243, Rv3616c:4055801, fluoroquinolone and pyrazinamide) were confirmed as important feature in lineage 1. An additional seven mutations (Rv3885c:4367649, Rv3614c:4054637, Rv3450c:3871333, Rv3876:4354013, Rv3450c:3871093, Rv3885c:4367090 and rifampicin) were tentative features, while the remaining mutations were rejected features.

Similarly, In **Figure 3b**, indicates that 14 mutations (Rv3445c:3863138, Rv2346c:2626018, Rv0290:353309, Rv3884c:4366195, Rv3620c:4060588, Rv0292:356528, Rv3879c:4357597, Rv0290:353365, ethambutol, streptomycin, fluoroquinolone, pyrazinamide, rifampicin, and isoniazid) were confirmed as important feature in lineage 2. Two additional mutations (Rv3879c:4359653, Rv3879c:4357804) were tentative features, while the remainder were rejected features.

In **Figure 3c**, two mutations (streptomycin and isoniazid) were confirmed as important features in lineage 3. One additional mutation (Rv3879c:4359165) was tentative feature, while the remainder were rejected features.

Finally, **Figure 3d** shows that 22 mutations (Rv3876:4353557, Rv3882c:4362807, Rv3864:4340330, Rv1783:2019942, Rv3616c:4055993, Rv3878:4357123, Rv3884c:4366195, Rv3879c:4357597, Rv3447c:3866350, Rv3884c:4365461, Rv3894c:4380236, Rv3874:4352475, Rv3879c:4359667, Rv3447c:3864995, Rv3882c:4363069, Rv3616c:4055801, streptomycin, pyrazinamide, ethambutol, rifampicin, fluoroquinolone and isoniazid) were confirmed as important features in lineage 4. Two additional mutations (Rv3882c:4362171 and Rv3447c:3867404) were tentative features, while the remainder were rejected features.

In addition, we conducted a detailed analysis of the L2 and L4 sublineages. As demonstrated in **Additional File 2**: **Supplementary Figures S8–S11**, mutations in L2.2.1, L4.1, L4.3 and L4.8 are a total of 22, 18, 14 and 10, respectively, which have been verified as follows:

L2.2.1: twelve mutations (Rv3445c:3863138, Rv2346c:2626018, Rv0290:353309, Rv3620c:4060588, Rv3879c:4357597, Rv3884c:4366195, ethambutol, streptomycin, pyrazinamide, rifampicin, fluoroquinolone and isoniazid) were confirmed. Three (Rv0290:353365, Rv0292:356528 and Rv3894c:4378504) mutations were tentative and the rest were rejected.

L4.1: 11 mutations (Rv3882c:4362807, Rv3879c:4359667, Rv3874:4352475, Rv3878:4357123, Rv3884c:4366195, streptomycin, pyrazinamide, rifampicin, ethambutol, isoniazid and fluoroquinolone) were confirmed and the rest were rejected.

L4.3: nine mutations (Rv1783:2019942, Rv3864:4340330, Rv3882c:4363069, streptomycin, fluoroquinolone, pyrazinamide, ethambutol, rifampicin and isoniazid) were confirmed. One (Rv3894c:4379680) was tentative and four were rejected.

L4.8: Two mutation (Rv3882c:4362171 and streptomycin) were confirmed and all other mutations were rejected.

These confirmed mutations have been identified as factors associated with genomic clusters.

#### 3.2.2 Risk mutations associated with genomic clusters selected by logistic regression analysis

To verify selected mutations with the Boruta algorithm, all 104 mutations were analyzed in univariate logistic regression analysis and any variable with a P value <0.2 in the univariate logistic regression analysis was included in the subsequent multivariate logistic regression analysis.

The analysis comprised 38 mutation sites in ESX genes in L1. Among the clustered and non-clustered strains of L1, 12 ESX gene mutation sites showed statistically significant differences (P<0.05), which showed these ESX gene sites were associated with the clustering of L1 when compared with non-clustered strains of L1(**Additional File 1**: **Supplementary Table S6**). Then, 14 mutation sites of ESX genes and three drug resistance genes with P<0.2 in univariate analysis were analyzed by multivariate regression. The results revealed that four mutations in ESX gene sites and two antimicrobial resistance mutations had a significant impact on the clustering of L1 (P<0.05), see **Additional File 1**: **Supplementary Table S12**, including two ESX mutation sites [espA (Rv3616c:4055801, OR, 5.053; 95% CI, 1.965-12.998), esxK (Rv1197:1340775, OR, 2.303; 95% CI, 1.134,4.680)] and one antimicrobial resistance mutation [fluoroquinolone (OR, 11.616; 95% CI, 2.420-55.769)] that were identified as risk factors for clustering.

The analysis focused on 16 ESX gene mutation sites in L2. In the comparison between clustered and non-clustered strains of lineage2, the difference in the mutation of nine ESX gene sites was statistically significant (P<0.05). The specific results can be found in (**Additional File 1**: **Supplementary Table S7**). Subsequently, all the 9 ESX gene mutation sites and drug resistant sites with P<0.2 in univariate analysis were included in a multivariate regression analysis, which showed that five mutation sites and two antimicrobial resistance mutations that were identified as risk factors for clustering, see **Additional File 1**: **Supplementary Table S13**, including esxU (Rv3445c:3863138, OR, 1.566; 95% CI, 1.327-1.848), esxO (Rv2346c:2626018, OR, 1.224; 95% CI, 1.083-1.383), esxW (Rv3620c:4060588, OR, 6.170; 95% CI, 1.375, 27.686), espK (Rv3879c:4357597, OR, 9.249; 95% CI, 1.093, 78.251), eccC2 (Rv3894c:4378504, OR, 3.669; 95% CI, 1.360, 9.900), ethambutol (OR, 2.310; 95% CI, 1.884, 2.832) and streptomycin (OR, 1.576; 95% CI, 1.310, 1.897).

24 mutation sites of ESX genes in L3 were analyzed. In the comparison between clustered and non-clustered strains, four ESX gene mutation sites showed significant differences (P<0.05), as detailed in **Additional File 1**: **Supplementary Table S8**. Subsequently, five mutation sites of ESX genes and one antimicrobial resistance mutation with P<0.2 were included in multiple regression analysis, and the results showed that 3 mutation sites of ESX genes that were identified as risk factors for clustering, see **Additional File 1**: **Supplementary Table S14**, including esxO (Rv2346c:2626095, OR, 14.519; 95% CI, 1.966-107.210) and eccC4 (Rv3447c:3864565, OR, 2.089; 95% CI, 1.377-3.170) (**Additional File 1**: **Supplementary Table S13**).

We analyzed 26 mutation sites in L4. Comparing clustered and non-clustered strains, there were 15 mutations in ESX gene sites with statistical significance (P<0.05), as detailed in **Additional File 1**: **Supplementary Table S9**. Subsequently, 18 ESX mutation sites and 6 antimicrobial resistance mutations with P<0.2 were analyzed by multivariate regression, and finally seven ESX gene sites and two antimicrobial resistance mutations with significant influence on clustering were determined(P<0.05), as shown in **Additional File 1**: **Supplementary Table S15**. Four ESX gene sites and two antimicrobial resistance mutations were risk factors for clustering, which were espE (Rv3864:4340330; OR, 2.203; 95% CI, 1.749-2.775), espA (Rv3876:4353557; OR, 21.020; 95% CI, 11.903-37.120), eccC5 (Rv1783:2019942; OR, 1.630; 95% CI, 1.209-2.198), eccE1 (Rv3882c:4362807; OR, 1.579; 95% CI, 1.008-2.473), pyrazinamide (OR, 1.760; 95% CI, 1.374, 2.256) and streptomycin (OR, 1.450; 95% CI, 1.199, 1.753).

The same analysis of L2.2.1, L4.1, L4.3 and L4.8, as detailed in **Additional File 1**: **Supplementary Tables S10**, **S16**. There were six risk mutations in L2.2.1. They were esxU (Rv3445c:3863138, OR, 1.722; 95% CI, 1.413-2.098), esxO (Rv2346c:2626018, OR, 1.252; 95% CI, 1.105-1.419), eccA2 (Rv3884c:4366195, OR, 10.571; 95% CI, 1.309-85.350), eccC2 (Rv3894c:4378504, OR, 2.928; 95% CI, 1.144-7.493), ethambutol (OR, 1.918; 95% CI, 1.575-2.336) and streptomycin (OR, 1.363; 95% CI, 1.183-2.837). Significantly, the SNP at Rv2346c:2626018 was found to be P<0.05 in the multivariate logistic regression analysis for L2, while the P value was 0.055 for L2.2.1. Similarly, the SNP at Rv3884c:4366195 was shown to be statistically significant (P<0.05) in L2.2.1, although its P value was 0.08 in L2. Therefore, it is clear that these two SNPs (Rv3884c:4366195 and Rv2346c:2626018) are potential significant mutations, and further investigation should be conducted. Two risk mutations in L4.1. They were streptomycin (OR, 1.832; 95% CI, 1.575-2.336) and pyrazinamide (OR, 2.469; 95% CI, 1.383-4.408). Three risk mutations in L4.3. They were streptomycin (OR, 1.997; 95% CI, 1.448-2.754), fluoroquinolone (OR, 2.172; 95% CI, 1.430-3.299) and pyrazinamide (OR, 1.684; 95% CI, 1.119-2.535). One risk mutation [eccA2 (Rv3882c:4362171, OR, 1.725; 95% CI, 1.232-2.415)] in L4.8.

### 3.3 Sensitivity analysis

In the sensitivity analysis, the lineage 1, lineage 2, lineage 3 and lineage 4 data were divided into four groups and then reanalyzed using Boruta algorithm and ordinal regression analysis. As shown in **Table 1**, the first, second, third, and fourth group included non-clustered isolates, small clusters containing two isolates, medium clusters containing 3 to 9 isolates, and large clusters containing >9 isolates, respectively. Only the mutations with a P value <0.2 in the univariate logistic regression analysis was included in the ordinal regression analysis.

**Figure 5:**
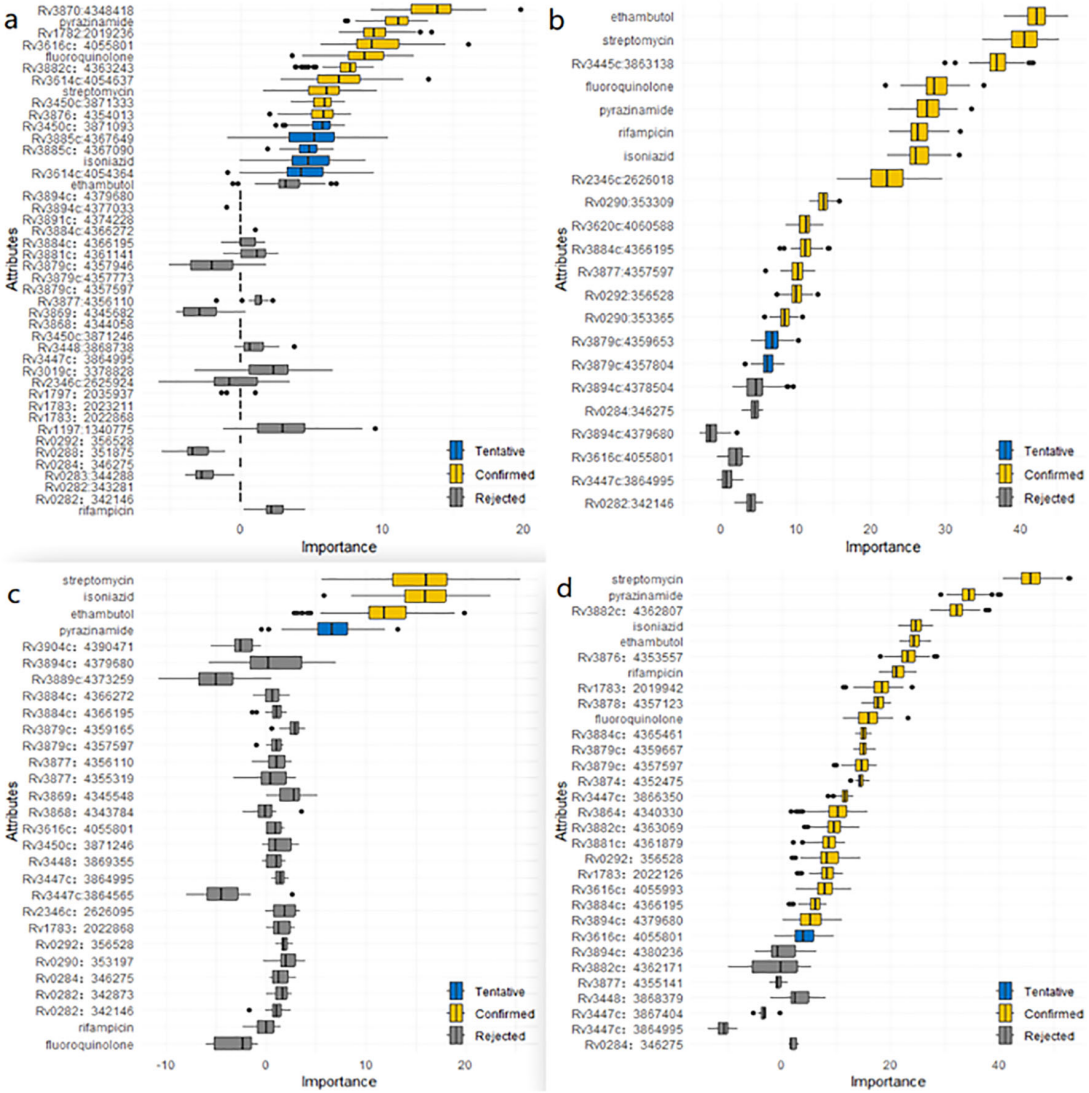
**(a)** Mutations associated with layered genomic clusters in ESX gene region of lineage 1 identified by the Boruta algorithm. **(b)** Mutations associated with layered genomic clusters in ESX gene region of lineage 2 identified by the Boruta algorithm. **(c)** Mutations associated with layered genomic clusters in ESX gene region of lineage 3 identified by the Boruta algorithm. **(d)** Mutations associated with layered genomic clusters in ESX gene region of lineage 4 identified by the Boruta algorithm.

The results of the Boruta algorithm were shown in **Figure 5**. As shown in **Figure 5a**, ten mutations (Rv3870:4348418, Rv1782:2019236, Rv3616c:4055801, Rv3882c:4363243, Rv3614c:4054637, Rv3450c:3871333, Rv3876:4354013, pyrazinamide, fluoroquinolone and streptomycin) were confirmed in lineage 1. Five mutations (Rv3450c:3871093, Rv3885c:4367649, Rv3885c:4367090, Rv3614c:4054364 and isoniazid) were tentative. The rest were rejected. As shown in **Figure 5b**, fourteen mutations (Rv3445c:3863138, Rv2346c:2626018, Rv0290:353309, Rv3620c:4060588, Rv3884c:4366195, Rv3879c:4357597, Rv0292:356528, Rv0290:353365, ethambutol, streptomycin, fluoroquinolone, pyrazinamide, rifampicin and isoniazid) were confirmed in lineage 2. Two mutations (Rv3879c:4359653 and Rv3879c:4357804) were tentative. The rest were rejected. As shown in **Figure 5c**, three mutations (streptomycin, isoniazid and ethambutol) were confirmed in lineage 3. One mutation(pyrazinamide) was tentative. The rest were rejected. As shown in **Figure 5d**, twenty-three mutations (Rv3882c:4362807, Rv3876:4353557, Rv1783:2019942, Rv3878:4357123, Rv3884c:4365461, Rv3879c:4359667, Rv3879c:4357597, Rv3874:4352475, Rv3447c:3866350, Rv3864:4340330, Rv3882c:4363069, Rv3881c:4361879, Rv0292:356528, Rv1783:2022126, Rv3616c:4055993, Rv3884c:4366195, Rv3894c:4379680, streptomycin, pyrazinamide, isoniazid, ethambutol, rifampicin and fluoroquinolone) were confirmed in lineage 4. One mutation (Rv3616c:4055801) was tentative. The rest were rejected. The results of ordinal regression analysis were shown in **Additional File 1**: **Supplementary Tables S17-S20**. There were six risk mutations in L1. They were eccB4 (Rv3450c:3871093, OR, 204944.568; 95% CI, 92661.384-453287.808), espA (Rv3616c:4055801, OR, 6.22; 95% CI, 2.327-16.623), espl (Rv3876:4354013, OR, 88974.512; 95% CI, 40227.955-196790.114), esxK (Rv1197:1340775, OR, 2.028; 95% CI, 1.005-4.093), espB (Rv3881c:4361141, OR, 514221.196; 95% CI, 232493.784-1137335.516) and fluoroquinolone (OR, 17.686; 95% CI, 4.299-72.765). There were six risk mutations in L2. They were esxU (Rv3445c:3863138, OR, 1.667; 95% CI, 1.42-1.958), esxW (Rv3620c:4060588, OR, 6.569; 95% CI, 1.457-29.607), eccC2 (Rv3894c:4378504, OR, 3.493; 95% CI, 1.279-9.538), espK (Rv3879c:4357597, OR, 9.102; 95% CI, 1.081-76.626) ethambutol (OR, 2.618; 95% CI, 2.158-3.175) and streptomycin (OR, 1.604; 95% CI, 1.346-1.911). The results showed that 2 mutation sites of ESX genes in L3 that were identified as risk factors for clustering, including esxO (Rv2346c:2626095, OR, 14.309; 95% CI, 1.938-105.672) and eccC4 (Rv3447c:3864565, OR, 2.29; 95% CI, 1.512-3.467). There were seven risk mutations in L4. These were espE (Rv3864:4340330; OR, 2.11; 95% CI, 1.692-2.631), eccE1 (Rv3882c:4362807; OR, 2.024; 95% CI, 1.307-3.137), eccC5 (Rv1783:2019942; OR, 1.404; 95% CI, 1.092-1.804), espA (Rv3876:4353557; OR, 35.653; 95% CI, 21.81-58.284), pyrazinamide (OR, 2.244; 95% CI, 1.801-2.797), streptomycin (OR, 1.412; 95% CI, 1.188-1.677) and ethambutol (OR, 1.491; 95% CI, 1.167-1.905).

As shown in **Figure 4**, the intersection results of the two independent methods were identified. Specifically, there was two risk mutations[espA(Rv3616c:4055801) and fluoroquinolone] in L1, five risk mutations [esxU(Rv3445c:3863138), esxW(Rv3620c:4060588), espK(Rv3879c:4357597), streptomycin and ethambutol], in L2, six risk mutations[espE(Rv3864:4340330), espl (Rv3876:4353557), eccE1(Rv3882c:4362807), eccC5(Rv1783:2019942), streptomycin and pyrazinamide] in L4. The sensitivity analysis results did not change significantly compared to those of the Boruta algorithm and multivariate regression analysis. The results of ordinal regression analysis based on the size of clustered isolates were like the main findings: one risk mutation[espA(Rv3616c:4055801)] in L1, four risk mutations [esxU(Rv3445c:3863138), esxO(Rv2346c:2626018), esxW(Rv3620c:4060588) and espK(Rv3879c:4357597)] in L2, four risk mutations[espE(Rv3864:4340330), espl (Rv3876:4353557), eccC5(Rv1783:2019942) and eccE1(Rv3882c:4362807)] in L4.

### 3.4 Deleterious effect of risk mutations on proteins

Nine SNPs[espA(Rv3616c:4055801), espK(Rv3879c:4357597), espE(Rv3864:4340330), espl(Rv3876:4353557), eccE1(Rv3882c:4362807), eccC5(Rv1783:2019942), esxU(Rv3445c:3863138), esxO(Rv2346c:2626018) and esxW(Rv3620c:4060588)] in the ESX gene region were predicted to negatively affect the respective proteins that affect the protein instability in nearby structural areas (**Table 2**).

**Table 2:** Deleterious effect of risk mutations on ESX proteins†.

Genomic position*	Nucleotide change	Amino acid change	stability
Rv3616c_4055801	G=>A	T192I	Large decrease of stability
Rv3879c_4357597	C=>G	C729S	Large decrease of stability
Rv3864_4340330	T=>G	L21V	Large decrease of stability
Rv3876_4353557	C=>T	P183L	Large decrease of stability
Rv3882c_4362807	A=>G	V205A	Large decrease of stability
Rv3445c_3863138	G=>A	P43S	Large decrease of stability
Rv2346c_2626018	T=>C	E52G	Large decrease of stability
Rv3620c_4060588	T=>C	T2A	Large decrease of stability
Rv1783_2019942	A=>G	Q229R	Large decrease of stability

†Functional impact of the risk mutations on protein structure and function was predicted on one protein prediction algorithms, I-Mutant v2.0 (http://folding.biofold.org/i-mutant/i-mutant2.0.html).

## 4 Discussion

From the analysis of genetic diversity, it was determined that the majority of these isolates belonged to lineage 4. While lineage 2 contributed a significant proportion, lineage 3 and lineage 1 were less prevalent. As illustrated in **Figure 1**, Lineage 2, also known as the East Asian lineage, including the Beijing family of strains, is primarily located in East Asia. However, it is also found in Central Asia, Russia, and South Africa. Lineage 4, also known as the Euro-American lineage, is frequently detected in individuals from Asia, Europe, Africa, and America. Lineages 1 and 3 were identified in regions in East Africa, South and Southeast Asia, Europe, and North America. On the other hand, lineages 5 and 7 are geographically more restricted and are generally confined to specific regions of Africa. These data are congruent with previous observations (Gagneux, 2007; Comas et al., 2009; Coscolla, 2014; Bañuls et al., 2015; Zheng et al., 2017; Gagneux, 2018; Koster et al., 2018).

Those risk mutations [espA(Rv3616c:4055801), espK(Rv3879c:4357597), espE(Rv3864:4340330), espl(Rv3876:4353557), eccE1(Rv3882c:4362807), eccC5(Rv1783:2019942), esxU(Rv3445c:3863138), esxO(Rv2346c:2626018) and esxW(Rv3620c:4060588)] were also predicted to negatively affect the respective proteins that affect the protein instability in nearby structural areas, supporting the hypothesis that they may affect TB transmission. Further research conducted through a range of biological and biochemical approaches has highlighted the critical role these genes play in Mycobacterium tuberculosis virulence. These findings have been confirmed in various animal and cellular models, demonstrating the fundamental role of these genes in Mycobacterium tuberculosis virulence (de Jonge et al., 2007; MacGurn and Cox, 2007; van der Wel et al., 2007; Smith et al., 2008; Sani et al., 2010; Abdallah et al., 2011; Houben et al., 2012; Simeone et al., 2012; Watson and Cox, 2012; Champion et al., 2014; Pajuelo et al., 2021). In addition, the findings of this study concur with previous genomic epidemiological articles (Mahairas et al., 1996; Hsu et al., 2003; Guinn et al., 2004; Wirth et al., 2012; Pang et al., 2013; Pajuelo et al., 2021).

The ESX protein complex plays a crucial role in the physiology, cell envelope integrity, conjugation, and host-pathogen interactions of mycobacteria. In order to evaluate the impact of mutations in the ESX gene on the global transmission of TB, a comprehensive analysis of 13582 strains of Mycobacterium tuberculosis, including 62 ESX genes, was performed. This study discovered ten risk mutations in the ESX gene regions that have the potential to boost TB transmission. These mutations are occurred in espA(Rv3616c:4055801), espK(Rv3879c:4357597), espE(Rv3864:4340330), espl(Rv3876:4353557), eccE1(Rv3882c:4362807), eccC5(Rv1783:2019942), esxU(Rv3445c:3863138), esxO(Rv2346c:2626018) and esxW(Rv3620c:4060588) gene region. EspA, espK, espE and espl are ESX-1 substrates. EccE1 is an essential component of the ESX-1 secretion system. esxU, esxO and esxW are ESAT-6 family members. EccC5 is predicted to be component of the ESX-5-membrane-associated complex.

ESX-1 is the prototype of type VII secretion systems (Hsu et al., 2003; Lewis et al., 2003; Pym et al., 2003), which is considered a major virulence factor of Mycobacterium tuberculosis through its essential role in phagosomal rupture and subsequent translocation of the pathogen to the host cytosol (Stamm et al., 2003; van der Wel et al., 2007; Houben et al., 2012; Simeone et al., 2012). Our study showed that five SNPs [espA(Rv3616c:4055801), espK(Rv3879c:4357597), espE(Rv3864:4340330), espl(Rv3876:4353557) and eccE1(Rv3882c:4362807)] in ESX-1 gene region were associated with clustering which could improve the TB transmission. EspA, espK, espE and espl are ESX-1 substrates which are virulence factors of Mycobacterium tuberculosis.

Research studies have indicated that disrupting disulfide bond formation within EspA could lead to a strain retaining ESX-1 function, while exhibiting significantly reduced virulence (Garces et al., 2010). Moreover, the secretion of EspA and EsxA is mutually dependent, with the deletion of espA resulting in the loss of EsxA secretion and subsequent attenuation (Fortune et al., 2005). A study also demonstrated that the espK gene is an essential player in preventing the formation of mature phagolysosomes and antigen presentation by host macrophages, and inhibiting espK expression could lead to a synergistic reduction in virulence and pathogenesis of Mycobacterium tuberculosis (Mishra et al., 2019).

The EspE substrate is found tethered to the mycobacterial cell surface and secreted into the extracellular environment *in vitro (* Carlsson et al., 2009; van der Woude et al., 2012). Recently, it has been reported that EspE is essential for the secretion of EsxA and plays a critical role in virulence within a macrophage infection model. Chirakos, Alexandra E et al. demonstrate that EspE is required for the lytic activity of the ESX-1 system and functions within the mycobacterial cytoplasm to negatively regulate the transcriptional activity of the WhiB6 protein, thereby modulating the production levels of ESX-1 substrates (Chirakos et al., 2020; Lienard et al., 2020).

EspI was not the subject of a comprehensive study. However, a limited body of research suggests that EspI may be involved in restraining the ESX-1-regulated secretion of bacteria when cellular ATP levels are low (Zhang et al., 2014). In addition, it is thought that the down-regulation of EspI may be critical for Mycobacterium tuberculosis’s ability to persist in chronic infections, as it maintains ATP levels and promotes virulence (Kruh et al., 2010; Zhang et al., 2014). The protein EccE1 is an indispensable component of the mycobacterial ESX-1 secretion system, which is crucial for the process of virulence factor secretion. EccE1 was initially believed to function as the inner membrane pore unit of a membrane complex, facilitating the transport of various substrates (Abdallah et al., 2007). In Mycobacterium tuberculosis, scientists discovered that the deletion of the eccE1 gene reduced the levels of EccB1, EccCa1 and EccD1, thereby abolishing ESX-1 secretion and reducing Mycobacterium tuberculosis ex vivo virulence (Soler-Arnedo et al., 2020). Additionally, in Mycobacterium smegmatis, the homolog of EccE1 was found to be required for the secretion of EsxA and EsxB (Converse, 2005). This indicates that EccE1 is critical for ex vivo virulence, stabilization of ESX-1 membrane proteins, and the secretion of EsxA, EsxB, EspA, and EspC. From the function of ESX-1substrates, a notable feature of the ESX-1 secretion system is the mutually dependent nature of protein export; i.e., the secretion of each substrate relies on the secretion of the other. Therefore, these SNPs could potentially modify the functionality of these proteins, subsequently impacting the secretion of one another and the overall virulence of Mycobacterium tuberculosis.

The Early Secreted Antigenic Target 6 (ESAT-6) family proteins are a group of small, spiral-structured proteins. These molecules are exported out of the cell via the ESX secretion system (Pallen, 2002). This family comprises a total of 23 members, labeled EsxA-W (Cole et al., 1998). These proteins have a pivotal role in host-pathogen interactions, serve as immunodominant antigens in the recognition of the human immune system, with the majority being immunodominant T cell antigens, playing a critical role in Mycobacterium tuberculosis pathogenesis and individual immune protection mechanism (Skjøt et al., 2000; Uplekar et al., 2011).

We found three SNPs [esxU(Rv3445c:3863138), esxO(Rv2346c:2626018) and esxW(Rv3620c:4060588)] in ESAT-6 family proteins have the potential to enhance the TB transmission. Previous research indicates that esxU can form a stable helical complex with esxT and has notable immunogenicities, characterized by significant lymphocyte proliferation and the induction of tumor necrosis factor-α (TNF-α) and interleukin-6 (IL-6) (Pandey et al., 2018). Currently esxW is an emerging vaccine candidate under investigation for inclusion in several TB vaccines (Baldwin et al., 2009; Bertholet et al., 2010; Knudsen et al., 2014; Baldwin et al., 2015), as it has demonstrated the ability to stimulate an immune response in mice, demonstrated safety and efficacy in non-human primates (Bertholet et al., 2010), and specifically targets T cells in humans (Bertholet et al., 2008). In one study, researchers identified an alteration in EsxW that could potentially contribute to enhanced transmission of Mycobacterium tuberculosis from the Beijing lineage (Holt et al., 2018). Lastly, the esxO gene in Mycobacterium tuberculosis is involved in virulence by enhancing survival within macrophages, by inhibiting the production of cytokines such as TNF-α and IL-6 (Mohanty et al., 2016; Yao et al., 2018).

The ESX-5 secretion system is critical for PPE protein secretion, cell wall stability and virulence; it is also critical for the uptake of nutrients across the outer membrane (Bottai et al., 2012; Ates et al., 2015). In this study, we discovered a single nucleotide polymorphism (SNP) in the EccC5 gene that might significantly elevate the odds of contracting Mycobacterium tuberculosis (Mtb). EccC5, a membrane-bound ATPase protein, is hypothesized to play a crucial role in the formation of the ESX-5 membrane-associated Mycobacterium tuberculosis complex. Ex vivo experiments have demonstrated that EccB5 and EccC5 encoding genes are parts of an operon and are indispensable for the survival and progression of Mycobacterium tuberculosis (Di Luca et al., 2012). The creation of a conditional mutant strain (MtbPptreccC5), in which the expression of the eccC5 gene was regulated by an anhydrotetracycline-repressible promoter, confirmed that eccC5 gene suppression is detrimental to the growth of Mycobacterium tuberculosis both *in vitro* and within human THP-1 macrophage cells (Di Luca et al., 2012). Further analysis of the secretome of Mycobacterium tuberculosis PptreccC5 strains revealed that EccC5 is required for the secretion of ESX-5-specific substrates, thus confirming that EccC5 is indeed a component of the ESX-5 secretion machinery (Di Luca et al., 2012). Moreover, a recent study has generated an M. marinum- Mycobacterium tuberculosis EccC5 chimera, demonstrating that the secretion specificity of PE_PGRS proteins in both M. marinum and Mycobacterium tuberculosis is reliant on the presence of EccC5 cognate linker 2 domain (Bunduc et al., 2020b).

In conclusion, this study provides statistical evidence that ten SNPs in Mycobacterium tuberculosis ESX genes may be associated with disease progression and increased transmission of certain strains. These SNPs may alter the protein’s function, further impacting adaptive responses by influencing the structure of nearby domains and triggering gene expression, ultimately influencing Mycobacterium tuberculosis transmission. It is important to note that while we have established the impact of these SNPs in ESX on the transmission of Mycobacterium tuberculosis, animal and immunological experiments should be conducted to gain further biological evidence and help us better comprehend the molecular biology characteristics of Mycobacterium tuberculosis. Research into the virulence of mycobacteria has demonstrated that the process of mutation is not primarily a result of the accumulation of nonsynonymous mutations, but more so, several crucial mutations that affect the activity of specific gene products (Hershberg et al., 2008; Mikheecheva et al., 2017). These proteins are produced by ten specific genes and are critical to the ability of the pathogen to survive in the host’s hostile environment. When these proteins are eliminated or deficient in biological experiments, Mycobacterium tuberculosis loses its virulence within the host (Fortune et al., 2005; Garces et al., 2010; Kruh et al., 2010; Di Luca et al., 2012; Zhang et al., 2014; Mohanty et al., 2016; Yao et al., 2018; Soler-Arnedo et al., 2020), highlighting the significant role that these proteins play in the pathogen’s life cycle. The findings from this study suggest that the ESX protein is a vital component of Mycobacterium tuberculosis ‘ life activities. Furthermore, this study offers several potential methods to intervene in the production or function of the ESX proteins, offering a wealth of potential targets for the design of novel antimycobacterial drugs. As such, the ten specific mutations identified in the ESX protein could potentially be considered as promising targets for future anti-tuberculosis therapies. Moreover, Due to the limitations of strain collection, the results of this study are only related to the whole set of strains used in the study.

## 5 Strength and limitations

This study presents several limitations. First, we did not perform animal and immunological experiments to provide biological validation for the risk mutations identified herein. Second, we lack critical host factors that may influence disease transmissibility, such as age, host immune status, and pulmonary cavitation. This absence hinders our ability to account for confounding variables that could elucidate the independent effects of risk mutations on transmissibility. Finally, contribution of analyzed strains is biased to one region (China). This has an impact in the biological inferences from a globally distributed human pathogen. In our subsequent research, we should endeavor to conduct more pertinent work. Of course, the sample size is large enough. The risk mutations we found were more reliable, which could provide credible data for TB prevention and treatment.

## Data Availability

The datasets presented in this study can be found in online repositories. The names of the repository/repositories and accession number(s) can be found in the article/**Supplementary Material**.

## Supplementary material

Supplementary Table 1 Information of 1445 strains of mycobacterium tuberculosis.

Supplementary Table 2 Information of 12137 strains of mycobacterium tuberculosis.

Supplementary Table 3 Information of gene mutations in ESX.

Supplementary Table 4 Six antimicrobial resistance mutations information of 13582 strains of mycobacterium tuberculosis.1 means resistant. 0 means sensitive.

Supplementary Table 5 SNP information of 13582 strains of mycobacterium tuberculosis in different countries.

Supplementary Table 6 Univariate regression analysis on SNPs associated with clustering in ESX gene region of lineage 1. Genomic position are genomic nucleotide positions in Mtb H37Rv genome NC_000962. Mutation isolates: In terms of SNPs, isolates that possess the mutation in the esx gene region are referred to as mutation isolates. The clustering rate was calculated as the percentage of cluster mutation isolates among total mutation isolates in L1 (number of cluster mutation isolates/number of total mutation isolates in L1). The mutation frequency was calculated as the percentage of mutation isolates among the number of total isolates in L1.

Supplementary Table 7 Univariate regression analysis on SNPs associated with clustering in ESX gene region of lineage 2. Genomic position are genomic nucleotide positions in Mtb H37Rv genome NC_000962. Mutation isolates: In terms of SNPs, isolates that possess the mutation in the esx gene region are referred to as mutation isolates. The clustering rate was calculated as the percentage of cluster mutation isolates among total mutation isolates in L2(number of cluster mutation isolates/number of total mutation isolates in L2). The mutation frequency was calculated as the percentage of mutation isolates among the number of total isolates in L2.

Supplementary Table 8 Univariate regression analysis on SNPs associated with clustering in ESX gene region of lineage 3. Genomic position are genomic nucleotide positions in Mtb H37Rv genome NC_000962. Mutation isolates: In terms of SNPs, isolates that possess the mutation in the esx gene region are referred to as mutation isolates. The clustering rate was calculated as the percentage of cluster mutation isolates among total mutation isolates in L3 (number of cluster mutation isolates/number of total mutation isolates in L3). The mutation frequency was calculated as the percentage of mutation isolates among the number of total isolates in L3.

Supplementary Table 9 Univariate regression analysis on SNPs associated with clustering in ESX g.

Supplementary Table 12 Multivariable regression analysis on SNPs associated with clustering in ESX gene region of lineage 1. Genomic position are genomic nucleotide positions in Mtb H37Rv genome NC_000962. * means that there is no result in statistical software or the result was too large and nonsense. OR, odds ratio.

Supplementary Table 13 Multivariable regression analysis on SNPs associated with clustering in ESX gene region of lineage 2. Genomic position are genomic nucleotide positions in Mtb H37Rv genome NC_000962.

Supplementary Table 14 Multivariable regression analysis on SNPs associated with clustering in ESX gene region of lineage 3. Genomic position are genomic nucleotide positions in Mtb H37Rv genome NC_000962.

Supplementary Table 15 Multivariable regression analysis on SNPs associated with clustering in ESX gene region of lineage 4. Genomic position are genomic nucleotide positions in Mtb H37Rv genome NC_000962.

Supplementary Table 16 Multivariable regression analysis on SNPs associated with clustering in ESX gene region of sublineages. Genomic position are genomic nucleotide positions in Mtb H37Rv genome NC_000962.

Supplementary Table 17 Ordinal regression analysis on SNPs associated with clustering in ESX gene region of lineage 1. Small clusters containing two isolates, medium clusters containing 3 to 9 isolates, and large clusters containing >9 isolates, respectively. Genomic position are genomic nucleotide positions in Mtb H37Rv genome NC_000962.

Supplementary Table 18 Ordinal regression analysis on SNPs associated with clustering in ESX gene region of lineage 2. Small clusters containing two isolates, medium clusters containing 3 to 9 isolates, and large clusters containing >9 isolates, respectively. Genomic position are genomic nucleotide positions in Mtb H37Rv genome NC_000962.

Supplementary Table 19 Ordinal regression analysis on SNPs associated with clustering in ESX gene region of lineage 3. Small clusters containing two isolates, medium clusters containing 3 to 9 isolates, and large clusters containing >9 isolates, respectively. Genomic position are genomic nucleotide positions in Mtb H37Rv genome NC_000962.

Supplementary Table 20 Ordinal regression analysis on SNPs associated with clustering in ESX gene region of lineage 4. Small clusters containing two isolates, medium clusters containing 3 to 9 isolates, and large clusters containing >9 isolates, respectively. Genomic position are genomic nucleotide positions in Mtb H37Rv genome NC_000962.

Supplementary Figure 1
**(a)** The phylogenetic tree analysis of lineage1.1. **(b)** The phylogenetic tree analysis of lineage1.2.

Supplementary Figure 2
**(a)** The phylogenetic tree analysis of lineage2.2.1. **(b)** The phylogenetic tree analysis of lineage2.2.2.

Supplementary Figure 3
**(a)** The phylogenetic tree analysis of lineage3. **(b)** The phylogenetic tree analysis of lineage3.1.

Supplementary Figure 4
**(a)** The phylogenetic tree analysis of lineage4.1. **(b)** The phylogenetic tree analysis of lineage4.2. **(c)** The phylogenetic tree analysis of lineage4.3. **(d)** The phylogenetic tree analysis of lineage4.4. **(e)** The phylogenetic tree analysis of lineage4.8.

Supplementary Figure 5 The phylogenetic tree analysis of lineage5.

Supplementary Figure 6 The phylogenetic tree analysis of lineage6.

Supplementary Figure 7 The phylogenetic tree analysis of lineage7.

Supplementary Figure 8 Mutations associated with genomic clusters in ESX gene region of lineage 2.2.1 identified by the Boruta algorithm. The yellow color represents confirmed feature. The blue color represents tentative feature and the grey color represents rejected feature.

Supplementary Figure 9 Mutations associated with genomic clusters in ESX gene region of lineage 4.1 identified by the Boruta algorithm. The yellow color represents confirmed feature. The blue color represents tentative feature and the grey color represents rejected feature.

Supplementary Figure 10 Mutations associated with genomic clusters in ESX gene region of lineage 4.3 identified by the Boruta algorithm. The yellow color represents confirmed feature. The blue color represents tentative feature and the grey color represents rejected feature.

Supplementary Figure 11 Mutations associated with genomic clusters in ESX gene region of lineage 4.8 identified by the Boruta algorithm. The yellow color represents confirmed feature. The blue color represents tentative feature and the grey color represents rejected feature.

Supplementary Figure 12 The intersection results of Lineage 2.2.1. The grey color means reject in the Boruta algorithm. The blue color means tentative in the Boruta algorithm. The yellow color means confirm in the Boruta algorithm. MLRA was the abbreviation of Multivariate Logistic Regression Analysis. OLRA was the abbreviation of Ordinal Logistic Regression Analysis. If the circle connected with MLRA or OLRA, it means the SNPs were risk mutations in MLRA or OLRA.

Supplementary Figure 13 The intersection results of Lineage 4.1. The grey color means reject in the Boruta algorithm. The blue color means tentative in the Boruta algorithm. The yellow color means confirm in the Boruta algorithm. MLRA was the abbreviation of Multivariate Logistic Regression Analysis. OLRA was the abbreviation of Ordinal Logistic Regression Analysis. If the circle connected with MLRA or OLRA, it means the SNPs were risk mutations in MLRA or OLRA.

Supplementary Figure 14 The intersection results of Lineage 4.3. The grey color means reject in the Boruta algorithm. The blue color means tentative in the Boruta algorithm. The yellow color means confirm in the Boruta algorithm. MLRA was the abbreviation of Multivariate Logistic Regression Analysis. OLRA was the abbreviation of Ordinal Logistic Regression Analysis. If the circle connected with MLRA or OLRA, it means the SNPs were risk mutations in MLRA or OLRA.

Supplementary Figure 15 The intersection results of Lineage 4.8. The grey color means reject in the Boruta algorithm. The blue color means tentative in the Boruta algorithm. The yellow color means confirm in the Boruta algorithm. MLRA was the abbreviation of Multivariate Logistic Regression Analysis. OLRA was the abbreviation of Ordinal Logistic Regression Analysis. If the circle connected with MLRA or OLRA, it means the SNPs were risk mutations in MLRA or OLRA.
