# Supplementary figures and images for: Type VII secretion system gene mutations driving global mycobacterium tuberculosis transmission revealed by whole genomic sequence
Source: Front Cell Infect Microbiol. 2025 Jun 18;15:1573643. doi: 10.3389/fcimb.2025.1573643 (PMC12213627; doi:10.3389/fcimb.2025.1573643)

---

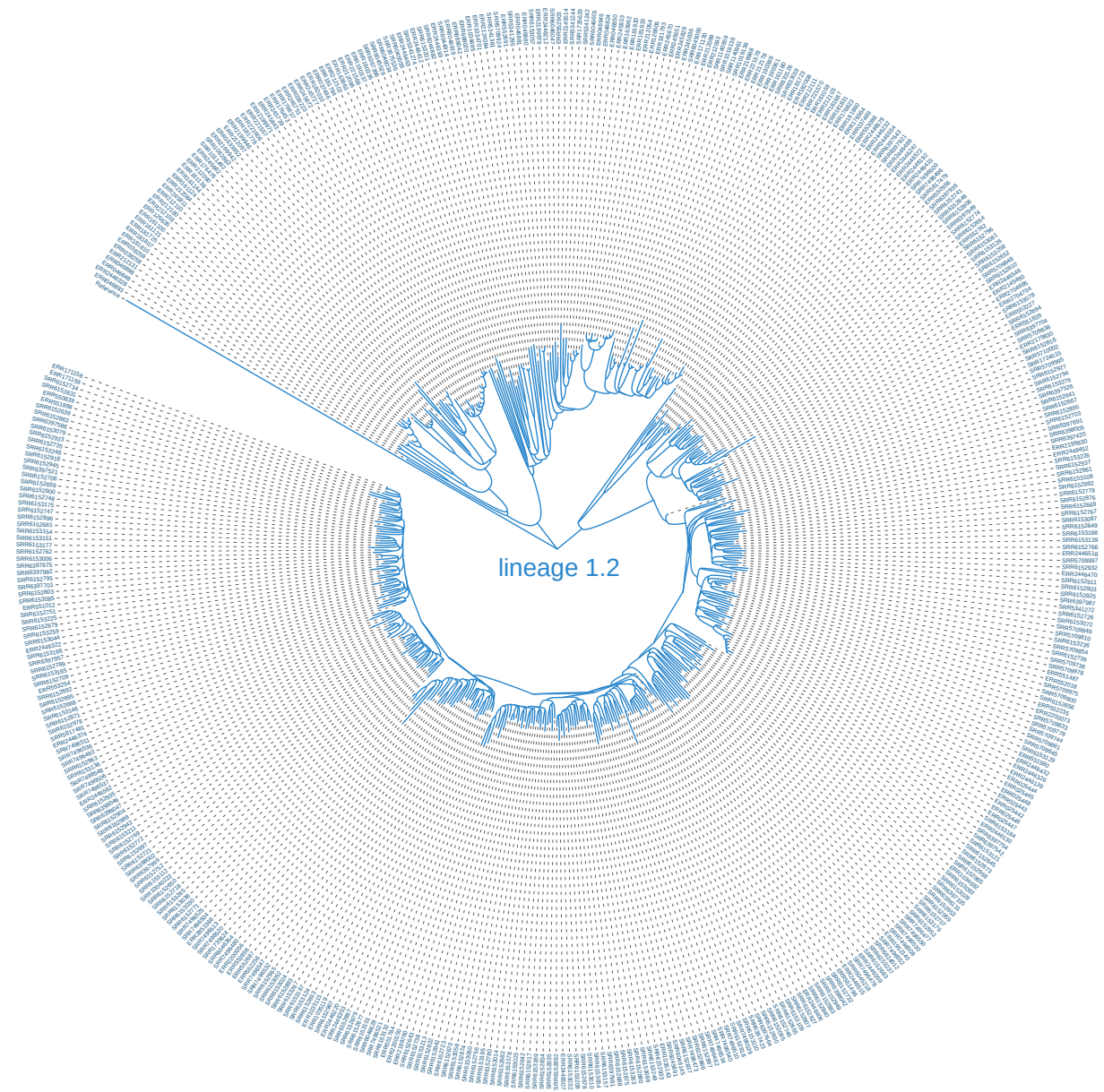

Supplement: Supplementary Figure 1 — (a) The phylogenetic tree analysis of lineage1.1. (b) The phylogenetic tree analysis of lineage1.2. [file Image1.pdf]

Tree scale: 0.0001

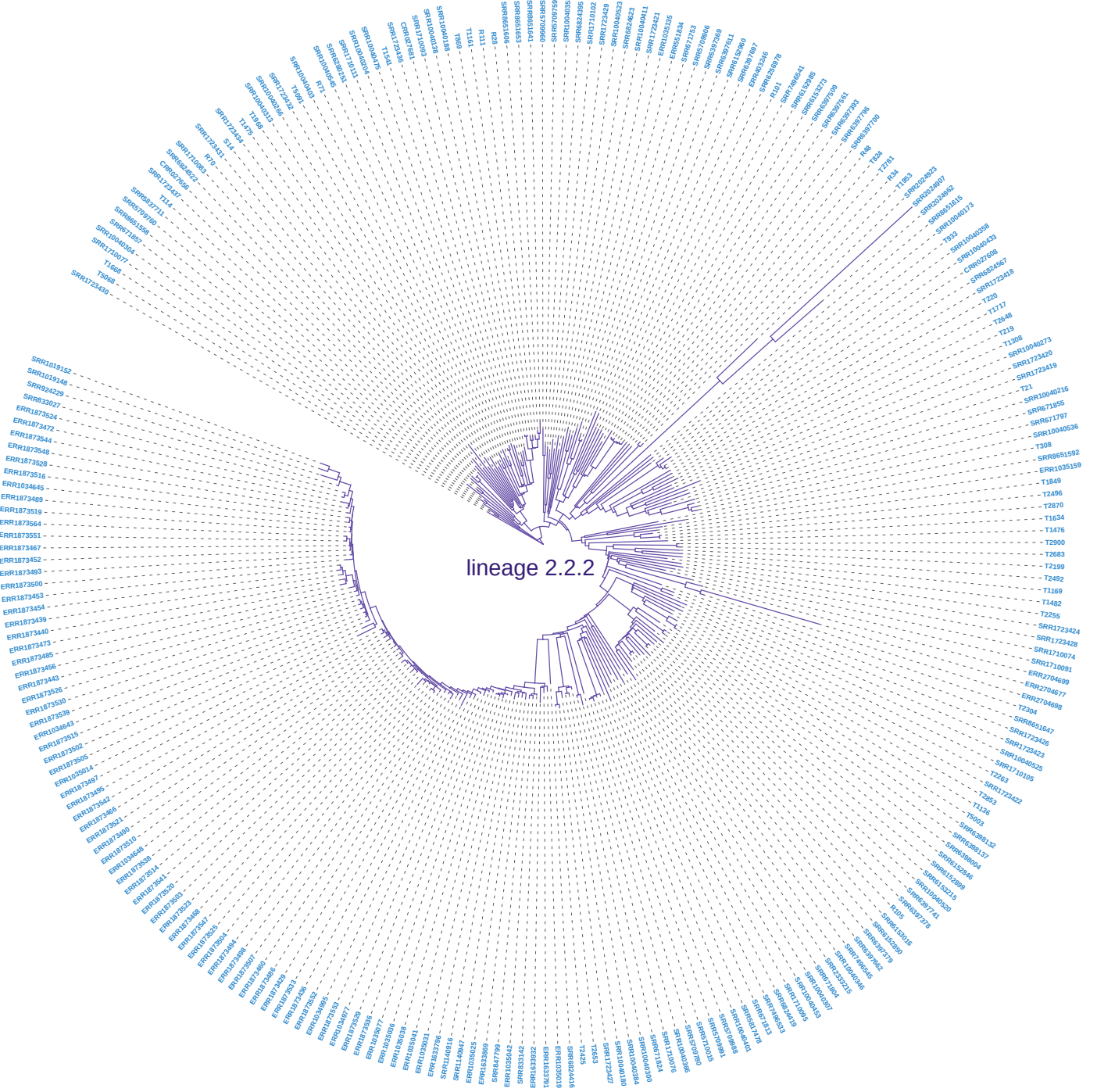

Supplement: Supplementary Figure 2 — (a) The phylogenetic tree analysis of lineage2.2.1. (b) The phylogenetic tree analysis of lineage2.2.2. [file Image2.pdf]

Tree scale: 0.0001

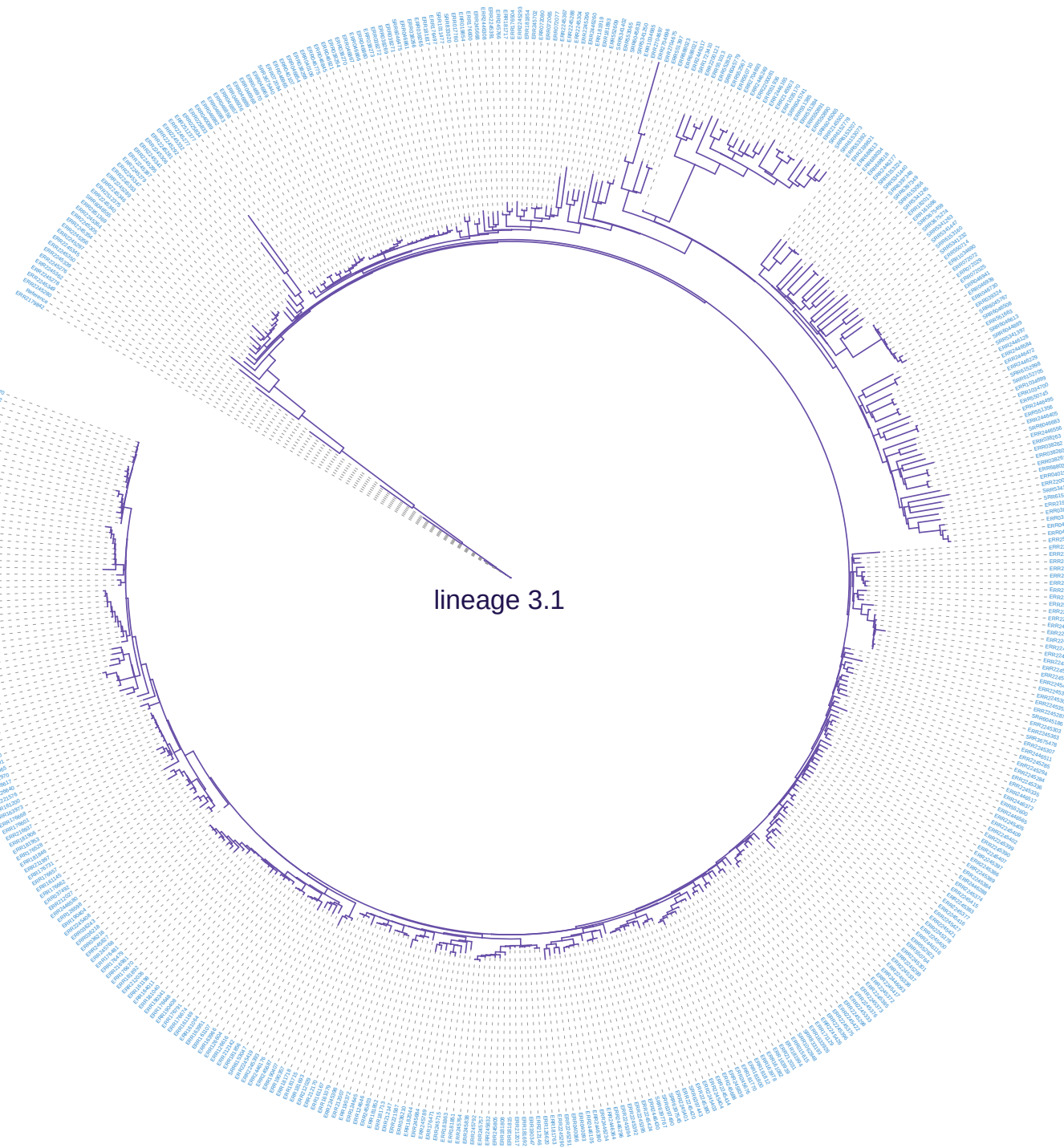

Supplement: Supplementary Figure 3 — (a) The phylogenetic tree analysis of lineage3. (b) The phylogenetic tree analysis of lineage3.1. [file Image3.pdf]

**Tree scale:** 0.0000099999999999999999 |-----|

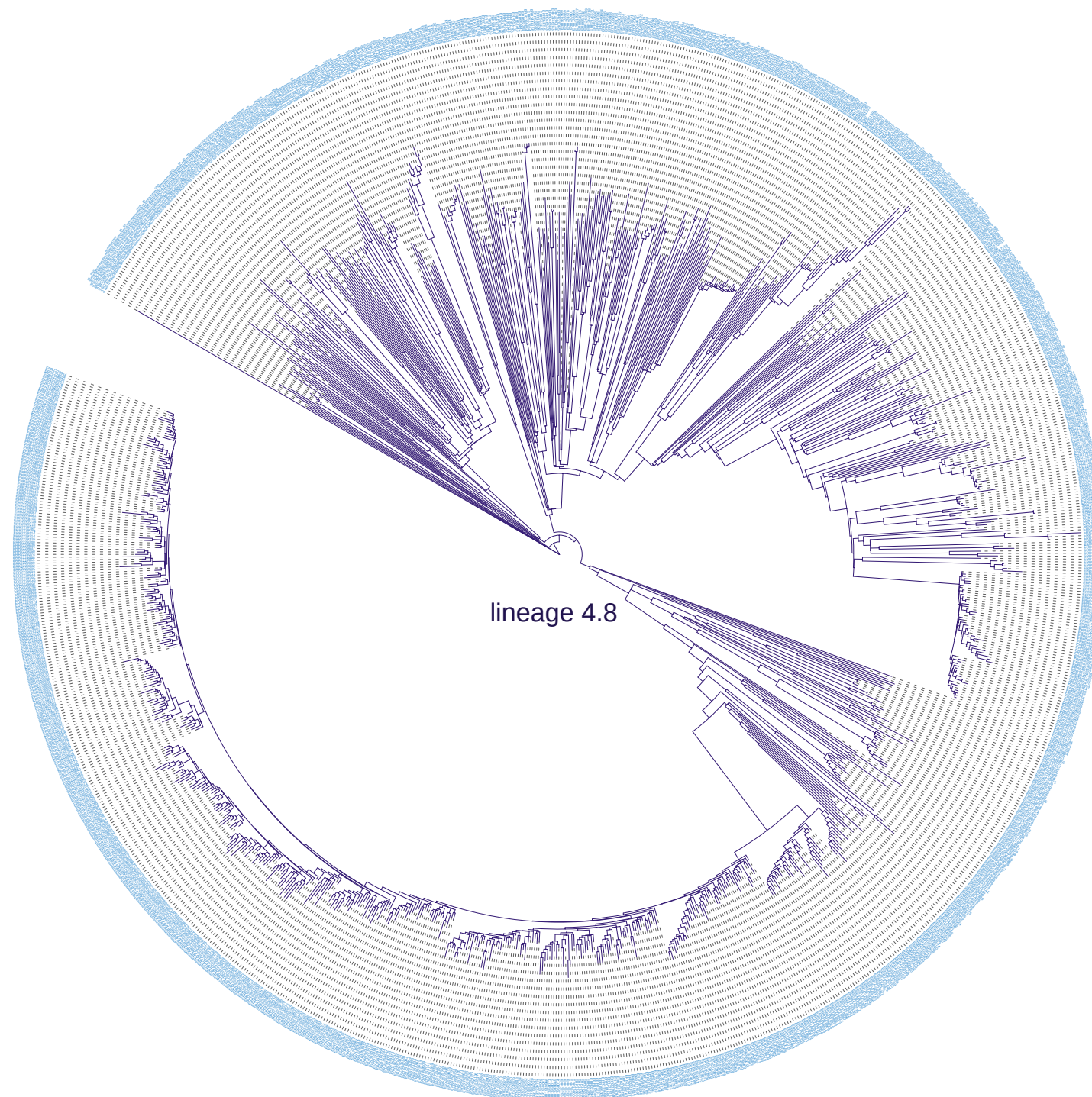

Supplement: Supplementary Figure 4 — (a) The phylogenetic tree analysis of lineage4.1. (b) The phylogenetic tree analysis of lineage4.2. (c) The phylogenetic tree analysis of lineage4.3. (d) The phylogenetic tree analysis of lineage4.4. (e) The phylogenetic tree analysis of lineage4.8. [file Image4.pdf]

Tree scale: 0.0001

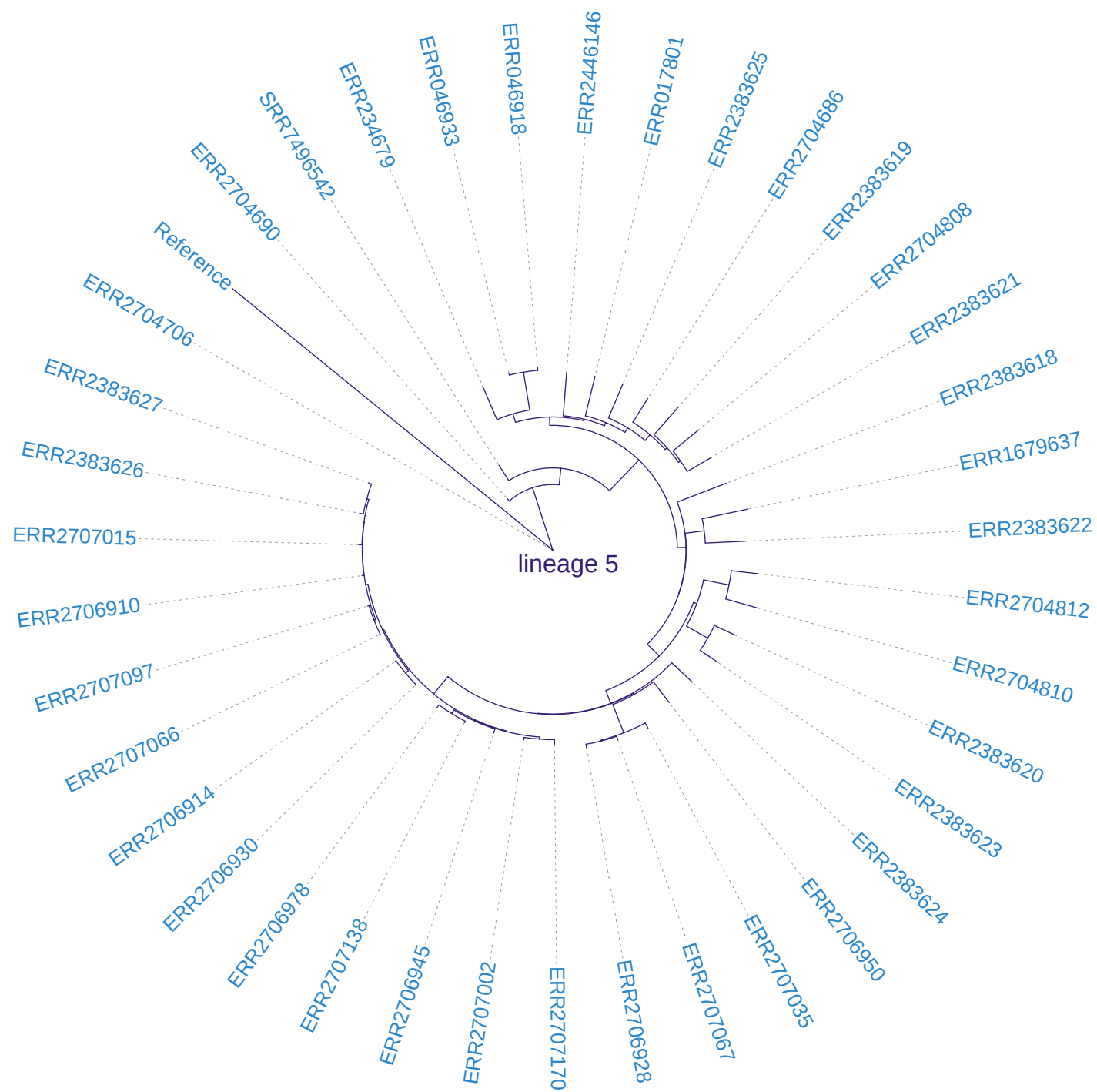

Supplement: Supplementary Figure 5 — The phylogenetic tree analysis of lineage5. [file Image5.pdf]

Tree scale: 0.0001

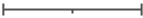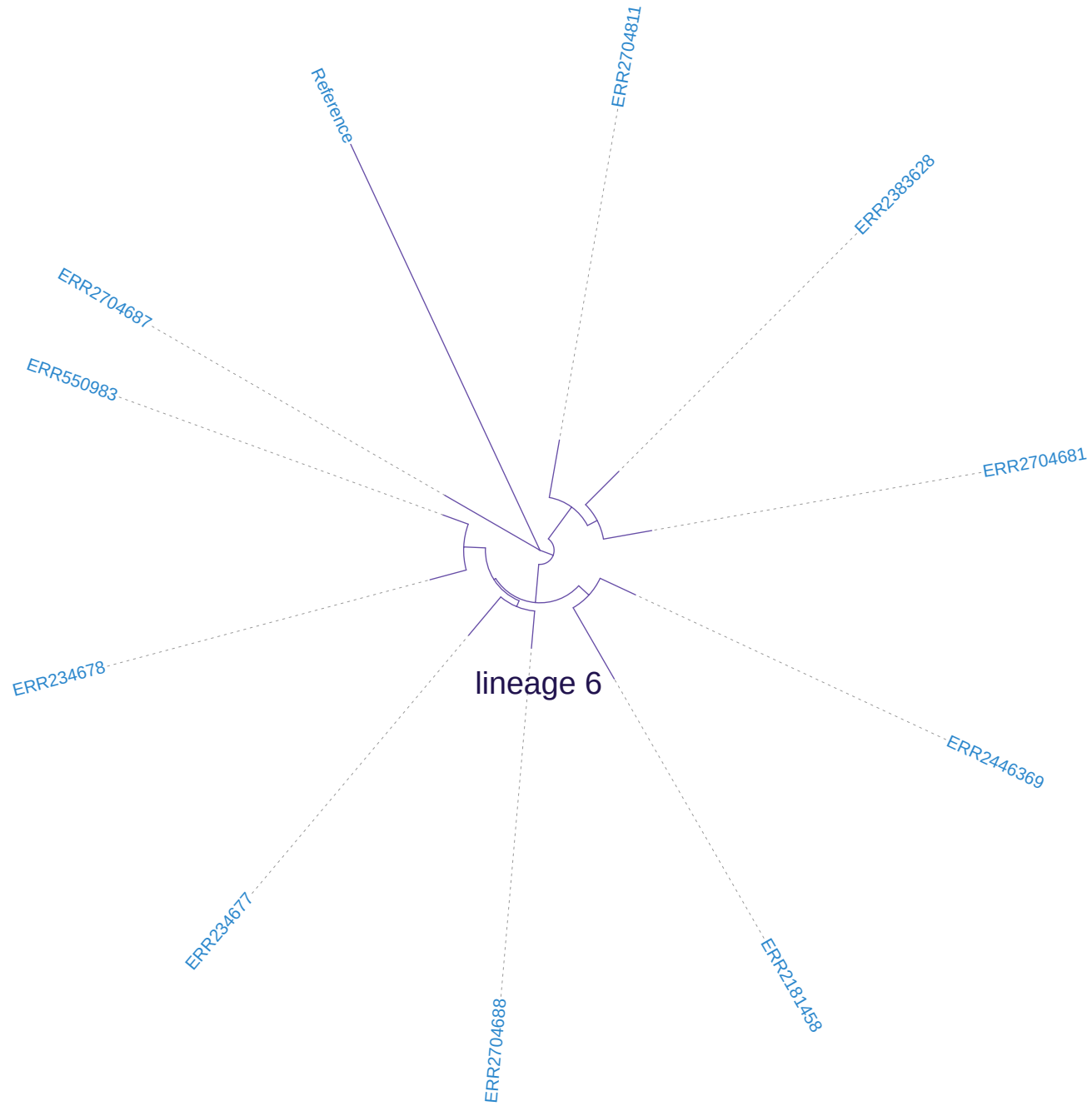

Supplement: Supplementary Figure 6 — The phylogenetic tree analysis of lineage6. [file Image6.pdf]

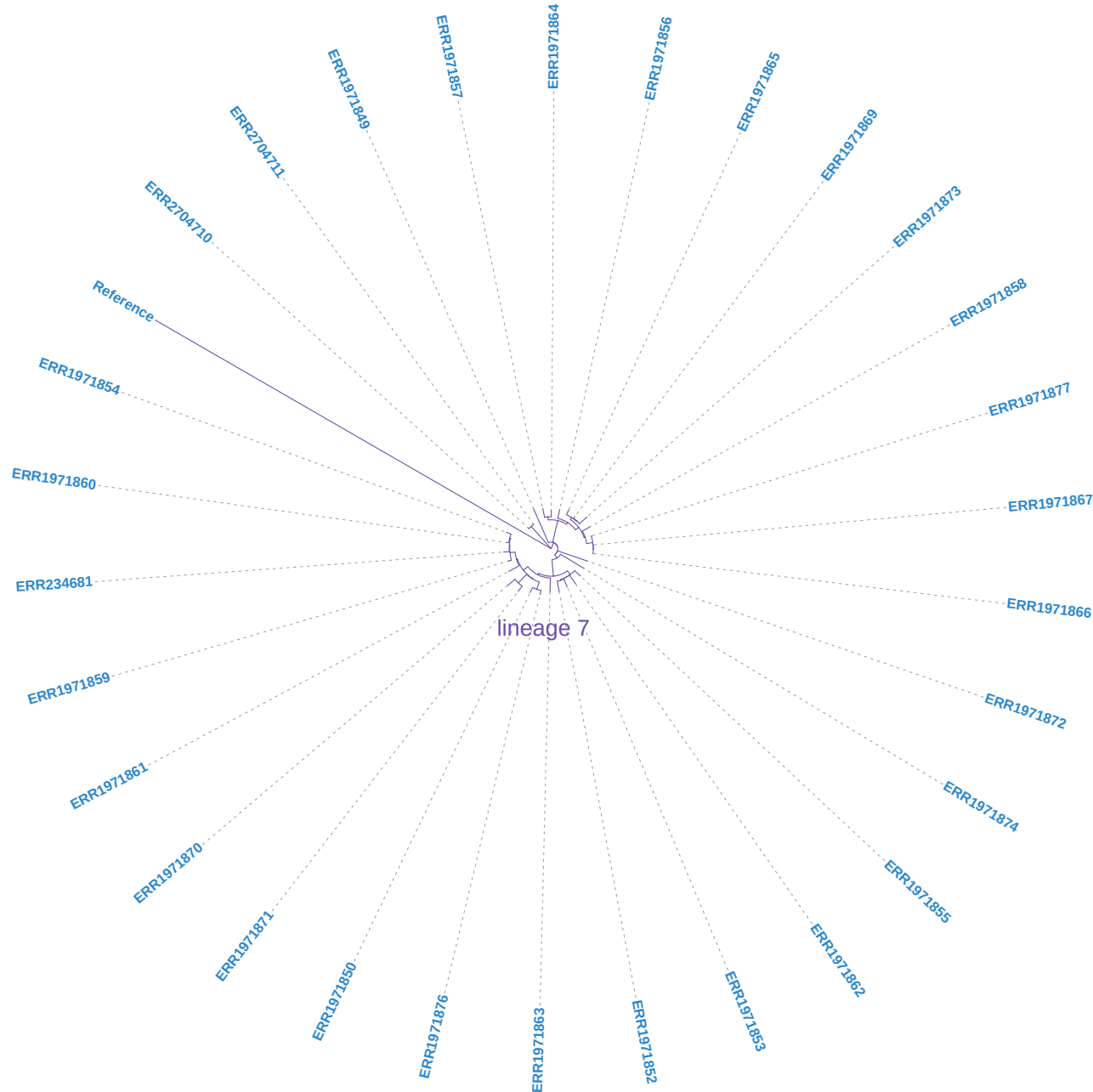

Supplement: Supplementary Figure 7 — The phylogenetic tree analysis of lineage7. [file Image7.pdf]

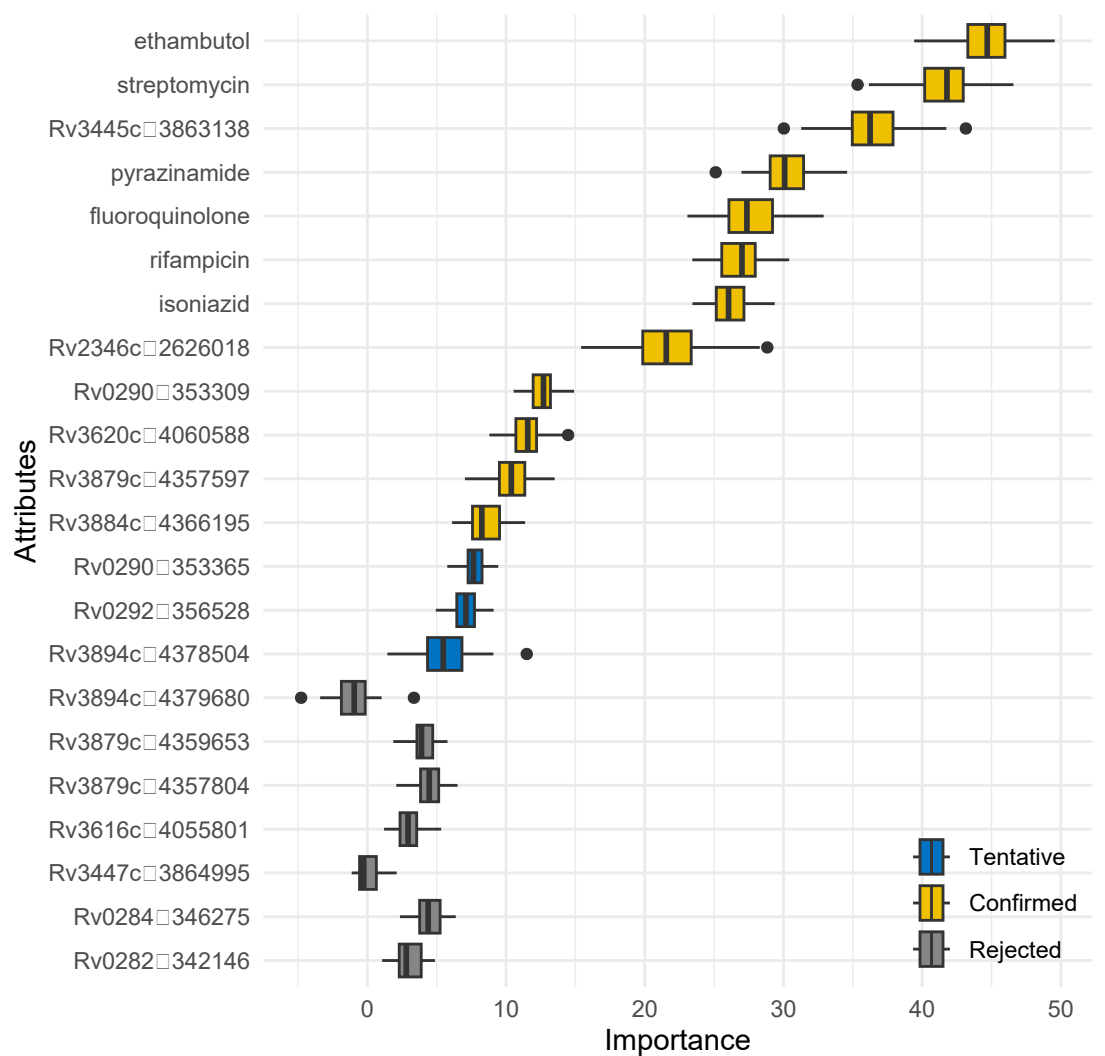

Supplement: Supplementary Figure 8 — Mutations associated with genomic clusters in ESX gene region of lineage 2.2.1 identified by the Boruta algorithm. The yellow color represents confirmed feature. The blue color represents tentative feature and the grey color represents rejected feature. [file Image8.pdf]

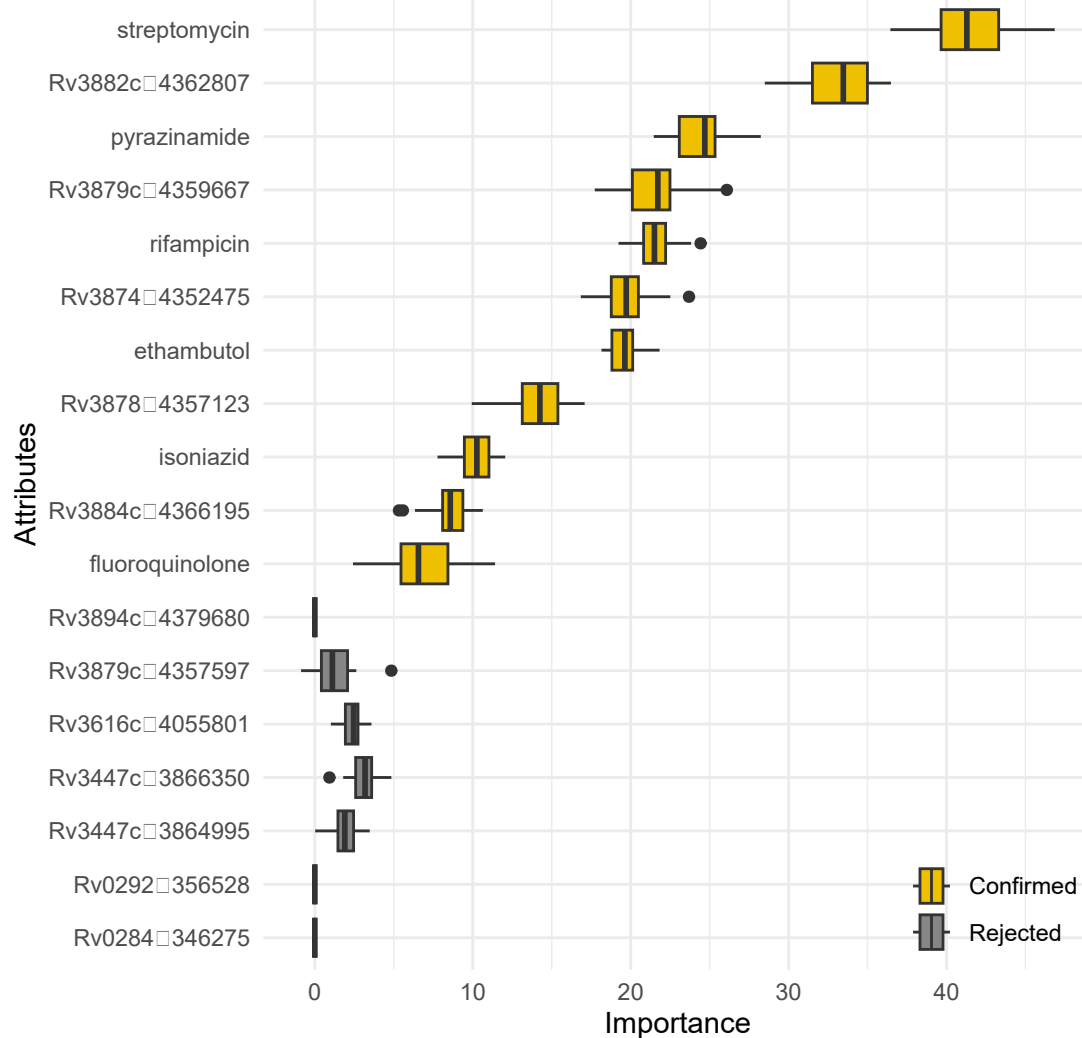

Supplement: Supplementary Figure 9 — Mutations associated with genomic clusters in ESX gene region of lineage 4.1 identified by the Boruta algorithm. The yellow color represents confirmed feature. The blue color represents tentative feature and the grey color represents rejected feature. [file Image9.pdf]

Attributes

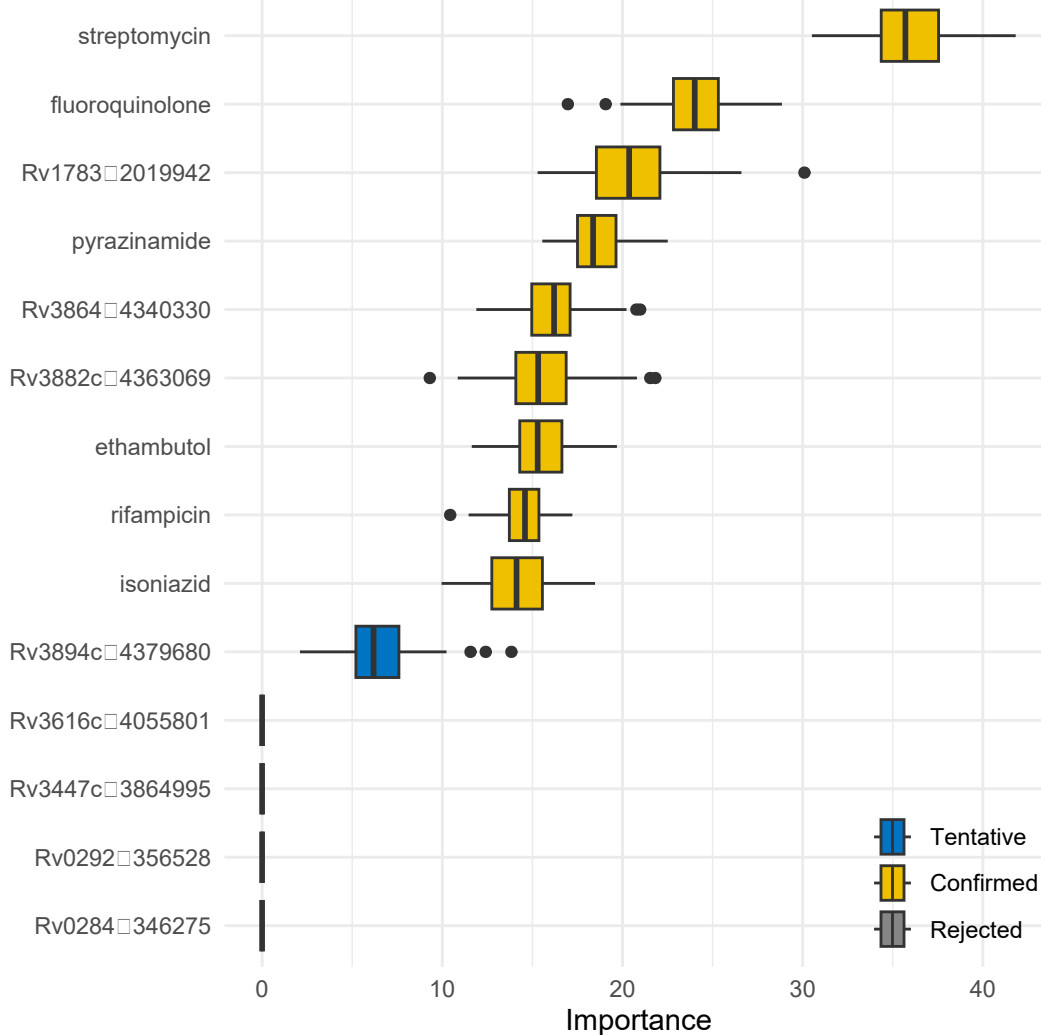

Supplement: Supplementary Figure 10 — Mutations associated with genomic clusters in ESX gene region of lineage 4.3 identified by the Boruta algorithm. The yellow color represents confirmed feature. The blue color represents tentative feature and the grey color represents rejected feature. [file Image10.pdf]

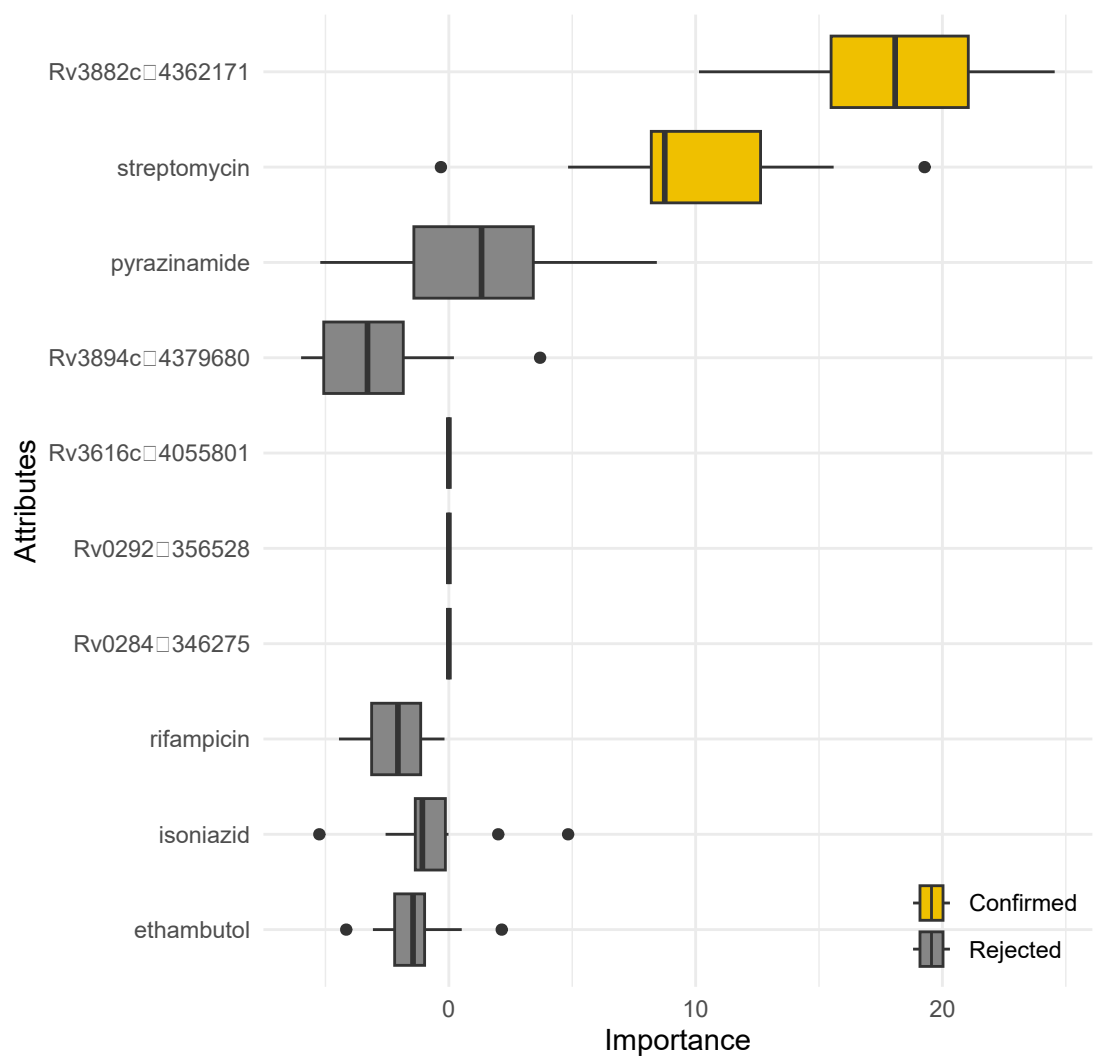

Supplement: Supplementary Figure 11 — Mutations associated with genomic clusters in ESX gene region of lineage 4.8 identified by the Boruta algorithm. The yellow color represents confirmed feature. The blue color represents tentative feature and the grey color represents rejected feature. [file Image11.pdf]

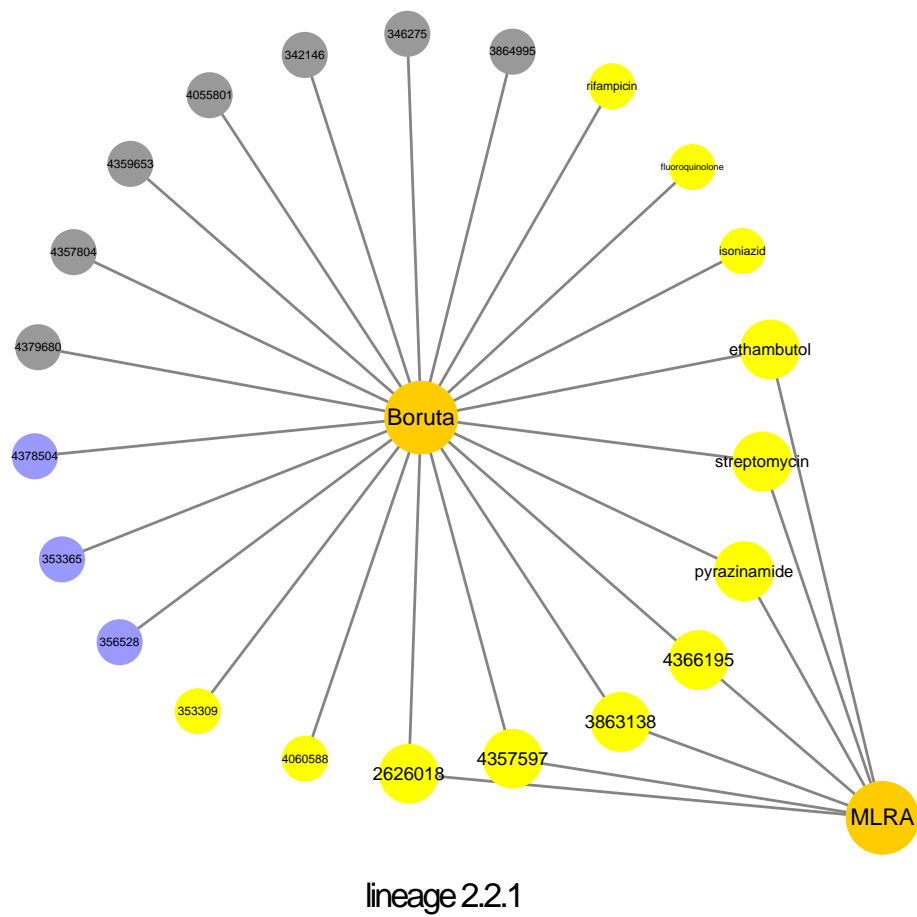

Supplement: Supplementary Figure 12 — The intersection results of Lineage 2.2.1. The grey color means reject in the Boruta algorithm. The blue color means tentative in the Boruta algorithm. The yellow color means confirm in the Boruta algorithm. MLRA was the abbreviation of Multivariate Logistic Regression Analysis. OLRA was the abbreviation of Ordinal Logistic Regression Analysis. If the circle connected with MLRA or OLRA, it means the SNPs were risk mutations in MLRA or OLRA. [file Image12.pdf]

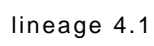

Supplement: Supplementary Figure 13 — The intersection results of Lineage 4.1. The grey color means reject in the Boruta algorithm. The blue color means tentative in the Boruta algorithm. The yellow color means confirm in the Boruta algorithm. MLRA was the abbreviation of Multivariate Logistic Regression Analysis. OLRA was the abbreviation of Ordinal Logistic Regression Analysis. If the circle connected with MLRA or OLRA, it means the SNPs were risk mutations in MLRA or OLRA. [file Image13.pdf]

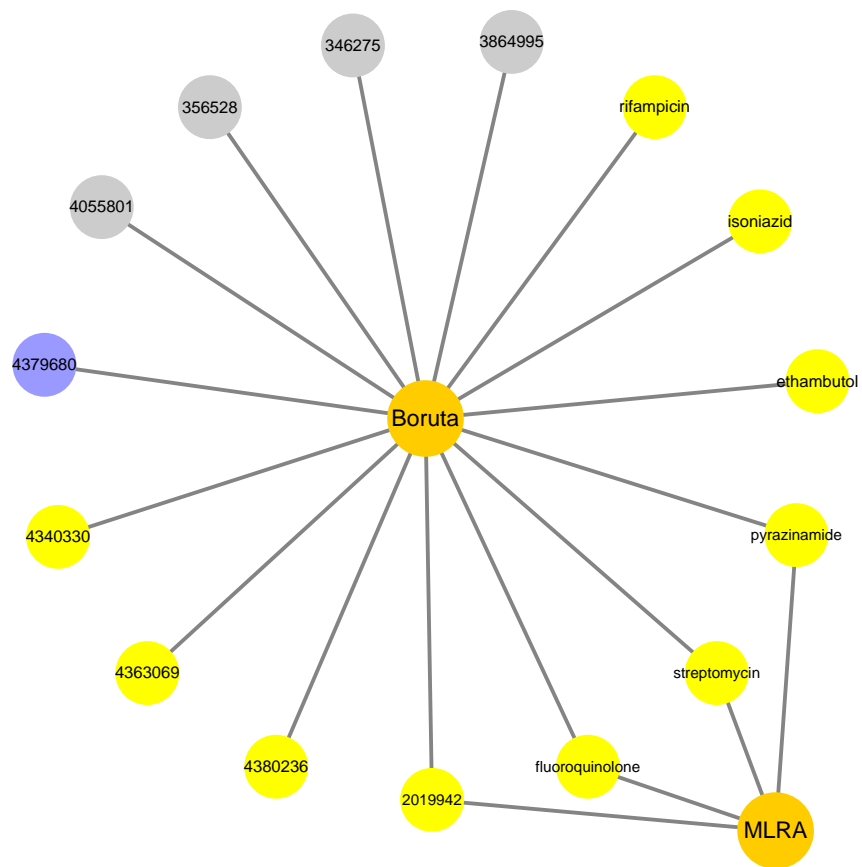

lineage 4.3

Supplement: Supplementary Figure 14 — The intersection results of Lineage 4.3. The grey color means reject in the Boruta algorithm. The blue color means tentative in the Boruta algorithm. The yellow color means confirm in the Boruta algorithm. MLRA was the abbreviation of Multivariate Logistic Regression Analysis. OLRA was the abbreviation of Ordinal Logistic Regression Analysis. If the circle connected with MLRA or OLRA, it means the SNPs were risk mutations in MLRA or OLRA. [file Image14.pdf]

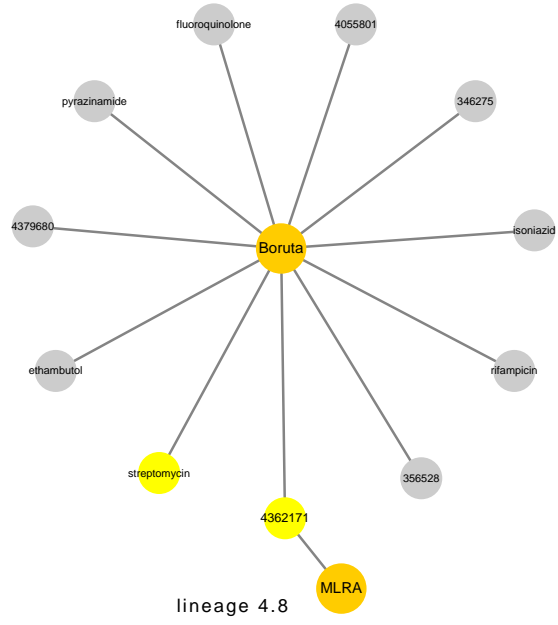

Supplement: Supplementary Figure 15 — The intersection results of Lineage 4.8. The grey color means reject in the Boruta algorithm. The blue color means tentative in the Boruta algorithm. The yellow color means confirm in the Boruta algorithm. MLRA was the abbreviation of Multivariate Logistic Regression Analysis. OLRA was the abbreviation of Ordinal Logistic Regression Analysis. If the circle connected with MLRA or OLRA, it means the SNPs were risk mutations in MLRA or OLRA. [file Image15.pdf]
